# Rice pseudomolecule-anchored cross-species DNA sequence alignments indicate regional genomic variation in expressed sequence conservation

**DOI:** 10.1186/1471-2164-8-283

**Published:** 2007-08-20

**Authors:** Ian Armstead, Lin Huang, Julie King, Helen Ougham, Howard Thomas, Ian King

**Affiliations:** 1 Institute of Grassland and Environmental Research, Plas Gogerddan, Aberystwyth, Ceredigion, SY23 3EB, UK

## Abstract

**Background:**

Various methods have been developed to explore inter-genomic relationships among plant species. Here, we present a sequence similarity analysis based upon comparison of transcript-assembly and methylation-filtered databases from five plant species and physically anchored rice coding sequences.

**Results:**

A comparison of the frequency of sequence alignments, determined by MegaBLAST, between rice coding sequences in TIGR pseudomolecules and annotations vs 4.0 and comprehensive transcript-assembly and methylation-filtered databases from *Lolium perenne *(ryegrass), *Zea mays *(maize), *Hordeum vulgare *(barley), *Glycine max *(soybean) and *Arabidopsis thaliana *(thale cress) was undertaken. Each rice pseudomolecule was divided into 10 segments, each containing 10% of the functionally annotated, expressed genes. This indicated a correlation between relative segment position in the rice genome and numbers of alignments with all the queried monocot and dicot plant databases. Colour-coded moving windows of 100 functionally annotated, expressed genes along each pseudomolecule were used to generate 'heat-maps'. These revealed consistent intra- and inter-pseudomolecule variation in the relative concentrations of significant alignments with the tested plant databases. Analysis of the annotations and derived putative expression patterns of rice genes from 'hot-spots' and 'cold-spots' within the heat maps indicated possible functional differences. A similar comparison relating to ancestral duplications of the rice genome indicated that duplications were often associated with 'hot-spots'.

**Conclusion:**

Physical positions of expressed genes in the rice genome are correlated with the degree of conservation of similar sequences in the transcriptomes of other plant species. This relative conservation is associated with the distribution of different sized gene families and segmentally duplicated loci and may have functional and evolutionary implications.

## Background

**Figure 1:**
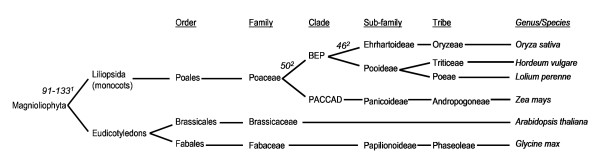
**Partial angiosperm taxonomy illustrating the relationship between monocot and dicot species included in the present analysis**. Numbers represent estimated times of lineage divergences (million years before the present) relative to rice taken from ^1^Bell et al. (2005) [26] and ^2^Gaut (2002) [58]. Taxonomic relationships were obtained from the NCBI Taxonomy browser [59].

In spite of evolutionary divergence and the pressures of domestication, there has been a noticeable conservation of genetic synteny between related plant species, e.g. within the Gramineae and the Brassicae. There has been considerable interest in defining these interrelationships, from the angles of both evolutionary genetics and plant breeding [1-8]. Rapidly accumulating data from plant genome sequencing and comparative genetic mapping have led to new resources for accessing and displaying these data sets (eg. see Gramene [9], The Institute for Genome Research (TIGR) [10], The Arabidopsis Information Resource (TAIR) [11], the Brassica Genome Gateway [12]. One limitation of the current state of knowledge in plant genetics is that the physically ordered complete genome sequence is only available for rice (*Oryza sativa*) and *Arabidopsis thaliana*, both of which have relatively small genomes. Rapid progress is being made for some other species, particularly maize (*Zea mays *[13,14], *Sorghum bicolor *[15,16] and *Brachypodium distachyon *[17] among the monocots and *Brassica *spp. [18] among the dicots (see also Joint Genome Initiative (JGI) [19]). However, feasible approaches for the sequencing and physical mapping of the larger genome Poaceae species, including the cereals wheat (*Triticum aestivum*) and barley (*Hordeum vulgare*) and the forage and amenity ryegrasses (*Lolium perenne *and *L. multiflorum*) and fescues (*Festuca pratensis *and *F. arundinacea*), are still in the process of development [20-23]. Until more progress is made in the whole genome analysis of these latter species, comparative studies will continue to be of great use in transferring information from the model species to the crop species. Typically, whole genome comparative studies are based upon the identification of genetic synteny between a model and crop species by either reference to existing sources or the development of *de novo *markers which target particular areas of a genome (see Gramene comparative map views [24]). In the present study a variation on the whole genome angle has been developed in which, using rice as an anchor, DNA sequence databases based upon both cDNA transcripts and methylation-filtration [25] obtained from both monocot and dicot crop and model species (see Figure 1 for a taxonomic description) have been aligned with the annotated rice pseudomolecules. This makes it possible to establish an overall picture of gene similarities between a number of different species and the rice genome.

## Results

**Figure 2:**
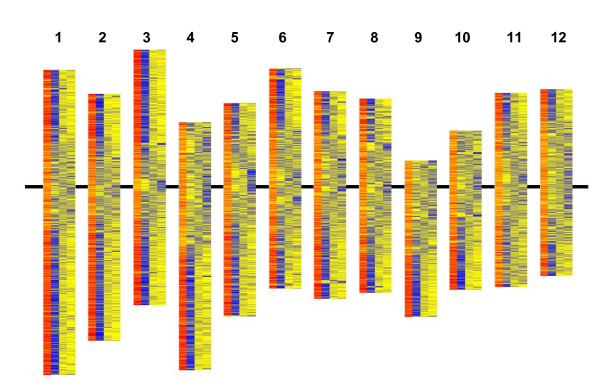
**Distribution of differently-annotated TIGR rice loci on rice pseudomolecules**. Linear order of differently annotated types of TIGR rice loci (TRL) on each of the rice pseudomolecules (1–12) in relation to significant MegaBLAST sequence alignments between the Os_CD database and the test databases. For each rice pseudomolecule: column 1 (red) = combined test database significant alignments; column 2 (blue) = functionally annotated TRL or expressed protein, column 3 (blue) = hypothetical protein, column 4 (blue) = retro/transposon-related sequence. Pseudomolecules are aligned along the centromere (horizontal black bar).

Using MegaBLAST with parameters of wordsize (W) = 16 and expectation (E) = 1 × 10^-10 ^identified significant alignments with between 7% (At_TA) and 45% (Zm_TA) of the all TIGR rice loci (TRL) (see Materials and Methods for database abbreviations). Subdividing the TRL database on the basis of annotation and pseudomolecule origin identified marked differences in the percentage of alignments within the different subdivisions [see Additional file 2 Table 1]. For all the databases, the largest percentage of alignments was identified for functionally annotated, expressed (FAexp) TRL (67% to 84% for the monocot databases and 21% to 28% for the dicot databases). The smallest number of alignments was identified in the hypothetical protein subdivision (7% to 10% for the monocot databases and < 0.05% for the dicot databases). These differences in the percentages of significant alignments coincided with differences in the physical distribution of the different types of TRL annotations (Figure 2). For each rice pseudomolecule the overall physical distribution of the TRL with functional annotations and the expressed proteins was distinguishable from the that of the hypothetical proteins and retro/transposon-related TRL. The distributions of the latter groups were loosely centred around the centromeres, whereas the former groups tended to be distributed away from the centromeres. The alignments established with the databases from the other species mimicked the distribution of the TRL with functional annotations and expressed proteins. On the basis of pseudomolecule origin, rice TRL derived from C3 generally had a higher percentage of alignments than those derived from the other pseudomolecules and TRL derived from C11 the lowest. This trend, in terms of percentages of alignments/pseudomolecule, was generally consistent across the different subdivisions of TRL and, particularly, the monocot plant databases [see Additional file 2 Table 1 and illustrated for FAexpTRL in Additional file 3]. An exception was the Lp_MF database, where the alignments from C1 and C3 were more or less equivalent.

**Table 1:** Spearman rank correlation coefficients between linear 10% FAexpTRL pseudomolecule segments comparing % MegaBLAST alignments and average scores for each test database.

Database		Lp_MF										
											
Lp_MF	a	a	Lp_MF									
												
Lp_MF	b	**0.465**	b	Zm_MF								
												
Zm_MF	a	**0.729**	**0.489**	a	Zm_MF							
												
Zm_MF	b	**0.630**	**0.672**	**0.577**	b	Zm_TA						
												
Zm_TA	a	**0.712**	**0.526**	**0.904**	**0.603**	a	Zm_TA					
												
Zm_TA	b	0.248**	**0.307**	**0.406**	**0.394**	**0.448**	b	Hv_TA				
												
Hv_TA	a	**0.711**	**0.495**	**0.756**	**0.554**	**0.818**	**0.450**	a	Hv_TA			
												
Hv_TA	b	0.256**	**0.391**	**0.479**	**0.462**	**0.522**	**0.809**	**0.456**	b	Gm_TA		
												
Gm_TA	a	**0.313**	**0.327**	**0.545**	0.275**	**0.603**	**0.566**	**0.550**	**0.550**	a	Gm_TA	
												
Gm_TA	b	*ns*	*ns*	*ns*	*ns*	*ns*	*ns*	*ns*	*ns*	*ns*	b	At_TA
												
At_TA	a	**0.320**	0.242**	**0.449**	0.259**	**0.490**	**0.620**	**0.509**	**0.056**	**0.702**	*ns*^1^	a
												
At_TA	b	*ns*	*ns*	*ns*	*ns*	*ns*	*ns*	*ns*	*ns*	*ns*	**0.672**	*ns*

a = % alignments

b = average scores

Significant levels: p < 0.001 (bold), ** = p < 0.01; * = p < 0.05; *ns *= p > 0.05

^1 ^p = 0.051

**Figure 3:**
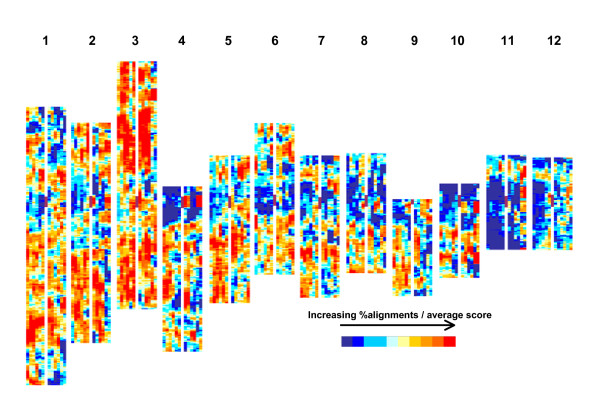
**Heat maps for % sequence alignments and average scores**. Colour coded moving windows/100 functionally annotated, expressed TIGR rice loci (MWs/FAexpTRL) for each rice pseudomolecule (1–12). For each pseudomolecule: column 1–6 = MWs for % significant MegaBLAST alignments between Os_CD and test databases Lp_MF, Zm_MF, Zm_TA, Hv_TA, Gm_TA and At_TA respectively; column 7 = position of MWs containing rice centromere (dark vertical bar); column 8–13 = MWs for average score of significant MegaBLAST alignments between Os_CD and test databases Lp_MF, Zm_MF, Zm_TA, Hv_TA, Gm_TA and At_TA, respectively [see Additional file 2 Table 3 for colour code quantification]. Pseudomolecule representations are aligned along the centromeres.

TIGR rice loci (TRL) with expressed functional annotations (FAexp) consistently generated the highest number of MegaBLAST alignments [see Additional file 2 Table 1]. Consequently these were considered likely to contain the most reliable gene predictions and further analyses were focussed on this set of 17108 gene models from the rice Os_CD database. Figure 2 illustrates how the FAexpTRL are not evenly distributed throughout the rice genome and while Figure 2 does not represent the direct physical distribution within each rice chromosome, it does reflect it. So, rather than using rice physical distances, in order to look at the patterns of alignments and their associated MegaBLAST scores, comparisons were made using linear 10% divisions of the FAexpTRL (n = 120, *i.e*. 10/pseudomolecule) and moving windows of 100 consecutive FAexpTRL (MWs) for each pseudomolecule (the number of MWs covering the 17108 FAexpTRL = 15920 – illustrated in Figure 3).

In comparing the results of the different test databases, the numbers of significant alignments and the scores associated with these alignments in each linear 10% of FAexpTRL for each pseudomolecule [see Additional file 2 Table 2] do not appear to be random, as indicated by the significant rank correlations (Table 1); *i.e*., the number of significant alignments identified for one test database for one pseudomolecule linear 10% segment is a positive indicator of the number of alignments one will expect to find in the other test databases. This is also true for comparisons between scores generated for the different test databases and for comparisons between numbers of alignments and scores (except for comparisons involving scores from the Gm_TA and At_TA databases) (Table 1). This trend can be illustrated using the MW approach (Figure 3) which shows 'hot' (red) and 'cold' (blue) spots relating to relative numbers of significant alignments and their scores – particularly for the four monocot test databases. Notably, FAexpTRL on Pm3 were aligned more frequently and those from Pm11 and Pm12 less frequently than FAexpTRL from the other pseudomolecules (Figure 3) [see Additional file 2 Table 2], though there is considerable local variation which is consistent particularly across the 4 monocot test databases. On most pseudomolecules, cold-spots were associated with the pericentromeric region, but were not exclusive to these regions.

**Table 2:** FAexpTRL blocks associated with high MegaBLAST scores from pseudomolecules 4 and 10

		Test database MegaBLAST score					
		
FAexpTRL	Annotation	Lp_MF	Zm_MF	Zm_TA	Hv_TA	Gm_TA	At_TA
LOC_Os04g16450	aquaporin PIP2.8, putative, expressed	470	357	351	838	121	121
LOC_Os04g16680	Sedoheptulose-1,7-bisphosphatase, chloroplast precursor, putative, expressed	289	995	1112	1112	184	129
LOC_Os04g16740	ATP synthase alpha chain, putative, expressed	1037	644	743	1126	856	815
LOC_Os04g16750	Photosystem I P700 chlorophyll a apoprotein A2, putative, expressed	613	868	644	628	470	462
LOC_Os04g16760	Photosystem I P700 chlorophyll a apoprotein A1, putative, expressed	503	1465	1100	323	357	252
LOC_Os04g16770	Photosystem Q, putative, expressed	936	190	539	1891	848	987
LOC_Os04g16780	Chloroplast 30S ribosomal protein S3, putative, expressed	129	287	474	498	97.6	141
LOC_Os04g16790	DNA-directed RNA polymerase alpha chain, putative, expressed	525	-	1086	1219	216	206
LOC_Os04g16819	DNA-directed RNA polymerase beta chain, putative, expressed	264	1265	410	394	105	188
LOC_Os04g16820	DNA-directed RNA polymerase beta chain, putative, expressed	1072	-	1096	460	266	-
							
LOC_Os10g21200	Photosystem Q, putative, expressed	936	1037	539	1883	840	979
LOC_Os10g21270	ATP synthase beta chain, putative, expressed	991	1524	1376	527	573	793
LOC_Os10g21280	Ribulose bisphosphate carboxylase large chain precursor, putative, expressed	936	591	1766	1701	531	981
LOC_Os10g21310	Photosystem II P680 chlorophyll A apoprotein, putative, expressed	561	287	551	170	206	-
LOC_Os10g21330	DNA-directed RNA polymerase alpha chain, putative, expressed	525	184	1070	1203	216	206
							
LOC_Os10g38229	Photosystem I P700 chlorophyll a apoprotein A1, putative, expressed	496	914	1092	323	357	252
LOC_Os10g38248	Photosystem I P700 chlorophyll a apoprotein A2, putative, expressed	1033	949	936	906	716	1076
LOC_Os10g38270	ATP synthase alpha chain, putative, expressed	1029	-	698	1098	848	815
LOC_Os10g38292	Chloroplast ATP synthase a chain precursor, putative, expressed	109	258	-	1096	545	614
Mean MegaBLAST score		214	260	504	473	155	151

- = no significant MegaBLAST alignment with Os_CD database

Figure 3 illustrates that the MWs based upon percentage numbers of alignments generally coincide with MWs based upon average scores. However, while the 2 data types are correlated (Table 1), the distributions do not exactly reflect each other and there are some notable regions of exception. For instance, the MWs in the centromeric region of Pm4 are relatively low, in terms of % alignments, have low relative MegaBLAST scores in the Hv_TA and Zm_TA databases, but have high relative MegaBLAST scores in the Lp_MF, Zm_MF, Gm_TA and At_TA databases. A similar patterns can be seen on Pm10 – with particularly high average scores derived for the Gm_TA and At_TA databases. Examination of the alignments in these regions of the pseudomolecule indicate this is caused by blocks of consecutive FAexpTRL aligned with each of the databases which have unusually high MegaBLAST scores (all 3 blocks also appear to have photosytem I and II functional associations; see Table 2). This becomes evident in some of the MegaBLAST scores-based MWs as opposed to the % alignments MWs, as the former are based on a relative quantitative measure, average score, whereas the latter are based on a qualitative, presence or absence, measure. In addition, while all the databases had unusually high MegaBLAST scores for the majority of the FAexpTRL described in Table 2, the relative increases/MW were larger in the Lp_MF, Zm_MF, Gm_TA and At_TA databases due to the lower, overall, average MegaBLAST scores for alignments from these databases. Also, the Lp_MF, Gm_TA and At_TA databases had lower overall numbers of alignments/MW [see Additional file 7].

**Figure 4:**
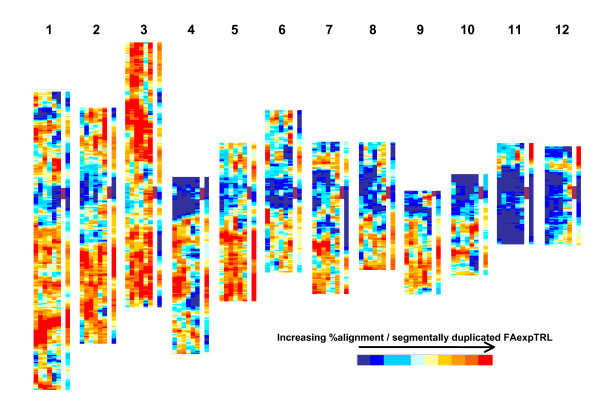
**Heat maps for % sequence alignments and segmentally duplicated rice loci**. Colour coded moving windows/100 functionally annotated, expressed TIGR rice loci (MWs/FAexpTRL) for each rice pseudomolecule (1–12). For each pseudomolecule: column 1–6 = MWs for % significant MegaBLAST alignments between Os_CD and test databases Lp_MF, Zm_MF, Zm_TA, Hv_TA, Gm_TA and AT_TA, respectively; column 7 = position of MWs containing rice centromere (dark vertical bar); column 8 = MWs indicating the distribution of segmentally duplicated FAexpTRL [see Additional file 2 Table 3 for colour code quantification].

Significant correlations between the number of segmentally duplicated FAexpTRL and % alignments/linear10% of each pseudomolecule (Table 3) indicated that there was a positive association between these measures. Illustrated in the form of MWs (Figure 4) it can be seen that regions where there are hot-spots or cold-spots for segmentally duplicated FAexpTRL also tend to be hot-spots or cold-spots for % alignments. Particular exceptions to this are on Pm11 and Pm12 where there are high concentrations of duplicated FAexpTRL but fewer alignments (omitting the equivalent FAexpTRL linear10% segments, 11.1, 11.2, 12.1 and 12.2 from the correlation calculation results in larger correlation coefficients, Table 3 [see Additional file 2 Table 1]. However, even in this case, the duplicated regions of the Pm11 and Pm12 have relatively more alignments than the remainders of these pseudomolecules.

**Table 3:** Spearman rank correlation coefficients between linear 10% FAexpTRL pseudomolecule segments comparing % MegaBLAST alignments for each of the test databases and the number of segmentally duplicated FAexpTRL

Test database	Segmentally duplicated FAexpTRL	
	A	B
Lp_MF	**0.532**	**0.592**
Zm_MF	**0.539**	**0.589**
Zm_TA	**0.516**	**0.586**
Hv_TA	**0.536**	**0.585**
Gm_TA	**0.351**	**0.373**
At_TA	0.185*	**0.256**

A = including pseudomolecule 11 and 12 segments 1 and 2 (n = 120).

B = excluding pseudomolecule 11 and 12 segments 1 and 2 (n = 116).

Significant levels: p < 0.001 (bold), * = p < 0.05;

[See Additional file 2 Table 2 for associated 10% FAexpTRL pseudomolecule 11 and 12 segments 1 and 2 values]

### Arabidopsis expression profiles

MegaBLAST searches of the 3057 FAexpTRL present in the highest 10% MWs (red colour code) [see Additional file 2 Table 3], which had significant alignments with all the monocot test databases, identified a total of 804 *Arabidopsis *gene models in the At_CD database (including hypothetical and expressed proteins). By searching the Genevestigator^® ^Meta-Analyzer database with these gene models, the expression profiles of 435 FAexp *Arabidopsis *gene models were obtained for both growth stage and plant organs, and the stage and organ of maximal expression were identified. The maximal expression profiles for these *Arabidopsis *gene models are compared with a random sample of 1599 FAexp *Arabidopsis *gene models in Tables 4 and 5.

**Table 4:** Maximal expression patterns according to organ type of *Arabidopsis *CDS from the At_CD database significantly aligned with FAexpTRL from the 'top 10%' alignment (red zone) regions of the pseudomolecules.

	'Top 10%' alignments		Random At_CD loci		
			
Plant Organ^1^	No.	% (A)	No.	% (B)	A-B
lateral root cap	35	8.0	53	3.4	4.6
callus	39	9.0	75	4.8	4.2
cell suspension	30	6.9	56	3.6	3.3
root tip	53	12.2	139	9.0	3.2
node	13	3.0	22	1.4	1.6
elongation zone	7	1.6	9	0.6	1.0
xylem	23	5.3	68	4.4	0.9
endodermis	13	3.0	35	2.3	0.7
endodermis + cortex	9	2.1	21	1.4	0.7
stele	8	1.8	24	1.5	0.3
shoot apex	16	3.7	54	3.5	0.2
rosette	1	0.2	1	0.1	0.1
root hair zone	23	5.3	81	5.2	0.1
influorescence	0	0	0	0	0
ovary	0	0	1	0.1	-0.1
cotyledons	9	2.1	34	2.2	-0.1
stem	2	0.5	10	0.6	-0.1
pedicel	7	1.6	28	1.8	-0.2
seedling	0	0	5	0.3	-0.3
adult leaf	3	0.7	16	1.0	-0.3
juvenile leaf	0	0	6	0.4	-0.4
epidermis atrichoblasts	12	2.8	49	3.2	-0.4
roots	1	0.2	10	0.6	-0.4
cork	7	1.6	33	2.1	-0.5
silique	4	0.9	23	1.5	-0.6
stigma	0	0	9	0.6	-0.6
stamen	11	2.5	49	3.2	-0.7
flower	0	0	10	0.6	-0.6
carpel	0	0	10	0.6	-0.6
petiole	1	0.2	16	1.0	-0.8
cauline leaf	8	1.8	41	2.6	-0.8
hypocotyl	3	0.7	25	1.6	-0.9
seed	14	3.2	66	4.3	-1.1
lateral root	6	1.4	38	2.5	-1.1
sepal	2	0.5	25	1.6	-1.1
hypocotyl	1	0.2	25	1.6	-1.4
petal	8	1.8	51	3.3	-1.5
radicle	0	0	25	1.6	-1.6
pollen	53	12.2	224	14.5	-2.3
senescent leaf	13	3.0	83	5.4	-2.4

Total	435		1550		

^1 ^Plant organs definitions as used in the Genevestigator™ database [57].

**Table 5:** Maximal expression patterns according to growth stage of *Arabidopsis *CDS from the At_CD database significantly aligned with rice FAexpTRL from the 'top 10%' alignments (red zone) regions of the pseudomolecules.

Growth stage^1 ^(days)	'Top10%' alignments		Random At_CD loci		
			
	No.	% (A)	No.	% (B)	A-B
1.0 – 5.9	86	19.7	187	11.8	7.9
6.0 – 13.9	28	6.4	86	5.4	1.0
14.0 – 17.9	36	8.2	167	10.5	-2.3
18.0 – 20.9	19	4.3	101	6.4	-2.1
21.0 – 24.9	99	22.7	258	16.3	6.4
25.0 – 28.9	43	9.8	180	11.4	-1.4
29.0 – 35.9	3	0.7	61	3.8	-3.1
36.0 – 44.9	33	7.6	140	8.8	-1.2
45.0 – 50	90	20.6	405	25.6	-5.0

Total	437		1585		

^1 ^Growth stages as described in the Genevestigator™ database [57].

## Discussion

It is well established that the physical and genetic order of markers and genes from one species can be reflected by similar orders or partial orders in a different species (synteny); a major goal of comparative plant genomics is to enable detailed analyses of these kinds to take place. It is also well established that detailed individual sequence alignments (*i.e*. as available through the TIGR and Gramene genome browsers) are informative in terms of establishing sequence similarities on a gene by gene basis. In contrast to these two approaches, this present study does not seek to comment directly on syntenic relationships between different plant species or to describe precise homologues or orthologues of individual genes. Additionally, it differs from other analyses in that the basic unit used is not a gene or a measure of physical, cytogenetic or genetic distance, but a unit of linearly ordered 'reliable' gene models for each pseudomolecule ('reliable' being defined as FAexpTRL for the purposes of this paper).

Six different databases, derived from 3 monocot species and 2 dicot species and generated either from expressed sequences or through methylation-filtered genomic DNA isolation, were tested against the Os_CD database. In the absence of the complete sequence of any genome there will always be limitations and unknowns in the degree of representation present in a given database. Additionally, the stringency of the cut-off threshold for reporting a significant alignment will, obviously, affect the outcome (E = 1 × 10^-10 ^in the present study). MegaBLAST wordsizes of 16, 20, 24, 28 (the MegaBLAST default) and 40 were tested with the Lp_MF database and these identified 63, 58, 48, 39 and 16% of the functionally annotated TRL, respectively. However, while the relative numbers of significant alignments identified was affected by altering the wordsize, the relative proportions of alignments between the different pseudomolecules stayed, more or less, the same [data not shown, but as illustrated for FAexpTRL in Additional file 3]. Consequently, the relatively permissive W = 16 was maintained in order to facilitate the identification of alignments, particularly in the context of searches of genomic (MF) databases against a database (Os_CD) which consists of predicted coding sequences.

There was clearly variation between the different test databases in the degree of coverage of the rice genome – particularly, and more obviously understandably, between the monocot and dicot databases, but also between the Lp_MF database and the other monocot databases. When compared directly with the other methylation-filtered database, while both the Lp_MF and Zm_MF databases contained similar numbers of sequences (Table 7) the average sequence length for Zm_MF was 752 bp as compared to 502 for Lp_MF. Additionally, 27% of the Zm_MF sequences were aligned with at least 1 TRL whereas the equivalent figure for the Lp_MF database was only 11%. These differences in the number of alignments identified between the Lp_MF database and the Zm_MF database probably relate more to the relative proportions and lengths of coding sequences present in the database than to sequence divergence between rice and ryegrass as compared to rice and maize.

The most striking aspect of the results presented here is the positional consistency of the similarities, in terms of identified alignments, between all the test databases – indicating that it is not just a consequence of random differences in sampling between the databases. This does not only apply to the monocot databases, but also to the dicot databases (Table 1, Figure 3) in spite of the relatively low number of overall alignments derived from the latter [see Additional file 2 Tables 1 and 2]. These positional relationships can even be seen at the level of the whole pseudomolecule: even though the magnitudes of the differences are not necessarily considerable, Pm11 consistently has the lowest number of alignments with the other databases and Pm3 (with the exception of the Lp_MF database where Pm3 and Pm1 have similar percentages) consistently has the highest number of alignments (Figure 3) [see Additional file 2 Tables 1 and 2]. Additionally, the average quality of alignments (as indicated by MegaBLAST scores) above the cut-off threshold also show positional similarities (Table 1, Figure 3). However, this is less consistent in terms of the MWs, chiefly because individual high scores can have a disproportionate effect on the overall average (*i.e*. the photosystem associated regions on Pm4 and Pm10 – see Results, Table 2). Assuming an ancestral monocot genome [26-28], the similarity of trends between the 4 monocot test databases and the rice Os_CD database would seem to indicate that there are positional differences in the rice genome in the rates at which coding sequences (or, at least, regions in coding sequences) evolve. Most notably, but not exclusively, Pm3 seems to contain many coding regions of the rice genome that have been relatively well conserved (in terms of sequence homology) with the other monocots tested. Conversely, Pm11 and Pm12 seem to contain many regions of the rice genome where the sequences of coding regions are less well conserved. These differences can, in part, be related to the relative distributions of FAexpTRL with unique annotations as opposed to FAexpTRL derived from large gene families (with family size determined from the annotation) [see Additional file 1 and particularly compare Pm3 with Pm11 in Additional file 4]. This observation must, however, be treated with caution due to the untested biological relevance of many annotations. Possibly, hot-spots represent genic regions of rice which have a more generalised function and cold-spots represent genic regions with more rice-specific functions. This is not to say that the homologues or orthologues of the genes present in rice on Pm11 and Pm12 and other cold-spots are absent from the other monocots – but it does suggest a greater degree of DNA coding sequence differences and so possible divergence in structure and function of the protein products from those regions of the genome. This may have implications in terms of using rice as a template for gene discovery and gene function prediction in the larger genome monocots.

There are few obvious differences between the 3 monocot test species in terms of the heat map patterns, though ryegrass is probably the most distinct. Bearing in mind the lower apparent coverage of the rice genome present in the Lp_MF database relative to the Zm_MF/TA and HV_TA databases, the hot-spots on Pm3 and Pm5 are less intense for ryegrass than they are for maize and barley (Figure 3). However, the hot-spots on Pm1 seem to be more intense for Lp_MF than for the other monocot databases. This would not be expected if it was just an artefact of the relative coverages of the different database. The relative number of hot-spots identified for Pm1 in the Lp_MF database is particularly interesting in the context of the high degree of conservation of genetic synteny that has been established between rice chromosome 1 and ryegrass/fescue (*Lolium/Festuca*) chromosome 3 [29].

In this analysis, there were 3057 FAexpTRL from Pm1, 2, 3, 4, 5, 7, 8, 9 and 10 which were in the top 10% (red zone) for the average monocot % alignment category and which also had significant alignments with all 4 monocot test databases (*i.e*. an average score of 1, see Materials and Methods). In comparison, there were 508 different FAexpTRL from Pm1, 2, 4, 6, 7, 8, 9 10, 11 and 12 which were in the bottom 10% (dark blue zone) for the average monocot % alignment category and which also had no significant alignments with any of the 4 monocot test databases. While it is not possible to draw any conclusions about the presence or absence of specific genes from either grouping, it is possible to look at the representation of large gene families (defined in this study as groups of FAexpTRL, with identical annotations, n > 100) within these top and bottom 10% groupings. Table 6 details that, of the 8 gene families which are represented > 100 times within the entire set of FAexpTRL, 4 out of the largest 5 families (protein kinase domain containing proteins, expressed; F-box domain containing proteins, expressed; leucine rich repeat (LRR) family proteins, expressed; NB-ARC domain containing proteins, expressed) seem to be over-represented in the bottom 10%. Three of these are also under-represented in the top 10% when compared to the overall average representation. Functionally, there is likely to be a degree of overlap between members of some of these families in that many F-box and NB-ARC domain containing proteins can also contain LRRs and kinase domains [30]. Clearly, these families are annotated on the basis of the presence of particular predicted structural motifs in the proteins, rather than by a well-developed knowledge of their precise functions. However, LRR and NB-ARC domain containing proteins are often associated with disease resistance and the hypersensitive response [30,31] and F-box proteins are associated with ubiquitin-targeting of proteins prior to degradation [32], therefore it is possible that the bottom 10% are enriched for genes with a rice-specific response to environmental challenges. In contrast, pentatricopeptide containing proteins are associated with plant organellar nucleotide metabolism and there is no inherent necessity for a rice-specific response [33,34]. More generally, within large gene families a degree of similarity in protein structure may allow for a degree of redundancy in protein function. This in turn would lead to a lessening of the relative degree of sequence homology in certain members of these gene families as species underwent divergent evolution (though, if this is a mechanism then it would appear not to apply to the pentatricopeptide gene family, which is slightly underrepresented in both the top and bottom 10%).

**Table 6:** Representation of FAexpTRL large gene families in the 'top' and 'bottom' 10% MWs

FAexp annotation large gene family^1^	Annotation type in all FAexpTRL		Annotation type in top^2 ^10% MWs		Annotation type in bottom^2 ^10% MWs	
			
	No.	% (n= 17108)^3a^	No.	% (n = 3057)^3b^	No.	% (n = 508)^3c^
Protein kinase domain containing protein, expressed	412	2.41	68	2.22	22	4.33
F-box domain containing protein, expressed	330	1.93	3	0.10	69	13.58
Leucine Rich Repeat family protein, expressed	318	1.86	20	0.65	50	9.84
pentatricopeptide, putative, expressed	264	1.54	16	0.52	1	0.20
NB-ARC domain containing protein, expressed	245	1.43	1	0.03	76	14.96
Zinc finger, C3HC4 type family protein, expressed	218	1.27	36	1.18	1	0.20
Cytochrome P450 family protein, expressed	184	1.08	22	0.72	8	1.57
RNA recognition motif family protein, expressed	117	0.68	16	0.52	0	0.00

^1^Large gene family = Families > 100 FAexpTRL with identical annotations.

^2^MWs which are in the top/bottom 10% for both % sequence alignments and average scores.

^3 ^n = a) total number of FAexpTRL; b) number of FAexpTRL in top 10% alignment MWs with significant alignments identified in each of the Lp_MF, Zm_MF, Zm_TA and Hv_TA (monocot) databases; c) number of FAexpTRL in bottom 10% MAWs with no significant alignments identified in any of the monocot databases.

A further question concerns whether there might be a biological basis associated with the observed heat-map patterns generated from the comparative alignment scores (Figure 3). To begin to address this, two further analyses were carried out relating to: a) possible expression patterns associated with FAexpTRL from the top10% (red zone) regions and b) the relation of the heat-map patterns generated from comparative alignments with that generated for segmentally duplicated FAexpTRL.

MegaBLAST identified 435 FAexp *Arabidopsis *gene models (for which expression profiles were present in the Genevestigator^® ^Meta-Analyzer) in the At_CD database with significant similarities to FAexpTRL and present in the top 10% (red zone) average monocot % alignment category. Considering just the plant organs (including callus and cell suspension; Table 4) or growth stages (Table 5) in which the FAexp *Arabidopsis *gene models were maximally expressed, when these were compared to a randomly selected set of FAexp *Arabidopsis *gene models there were some differences between the two samples in terms of the maximal expression profiles. In terms of the plant organs, the six types that appeared more frequently (on a percentage basis) in the top 10% group, as opposed to the control group, could all be associated directly or indirectly with meristematic cell division. Lateral root cap and callus tissue were particularly striking in this respect. For growth stage, those associated with germination and the initiation of floral development were represented more strongly in the top 10% group than in the control. No comparison could be made with FAexpTRL present in the bottom 10% as, by definition of the MegaBLAST discrimination used in this study, these were not represented within the At_CD database. There is an indication, from both the annotation comparisons and the maximal expression profile comparisons, that some genes within hot- and cold-spots may have a degree of diverged functionality. In support of this principle, growth stage differences in the overall transcription patterns between euchromatin and heterochromatin in rice have been noted previously for chromosome 4 [35]. Similarly, in the present study, there is a general association of cold spots and hot-spots with regions of the pseudomoleules with higher and lower concentrations, respectively, of retro/transposon related sequences (compare Figure 2 and Figure 3). This indicates that there is a hot-spot/euchromatin, cold spot/heterochromatin association. However, one must be extremely wary of over-interpretation and clarification of this will have to await further investigation.

There has been a considerable amount of genome duplication in the development of the modern-day rice genome, principally through ancestral polyploidisation, but also involving more recent duplications [27,36,37] (see TIGR, segmental genome duplication of rice [38]) and the consequences of gene duplication and the fates of such genes has been the topic of recent interest [39-47]. Genome duplication by polyploidisation in the modern rice progenitor is considered to have occurred c. 70 million years ago, before the divergence of the Poaceae cereal and grass genomes, and to have been followed by a diploidisation process involving large-scale chromosome rearrangement and deletion [27,37]. In this study, when the positions of segmentally duplicated regions of the rice genome were related to % sequence alignments within linear 10% FAexpTRL Pm segments, there was found to be a positive correlation for all of the test databases (Table 3). This relationship, illustrated in the equivalent MWs in Figure 4, indicates that the presence of hot-spots for segmentally duplicated FAexpTRL is usually associated with the presence of hot-spots for % alignments. It has been reported that paralogous gene pairs produced from whole genome duplications evolve more slowly than singletons [48,49] and the association of segmentally duplicated and % alignment FAexpTRL hot-spots is consistent with this. Models have been proposed as to why the conservation of both paralogous genes resulting from a duplication event might be maintained over evolutionary time. One such model posits subfunctionalisation, in which the combined activities of paralogous genes maintain the function(s) of their single common ancestor, though neither paralog, separately, maintains the complete original function [40,41]. It is also proposed that paralogs might provide protection against deleterious mutations in dosage sensitive genes, particularly those with significant roles in interactive gene networks [47,50]. It could be conjectured that the hot-spots in this study represent the physical position in the rice genome of plant genes which require more precise conservation of sequence in order to maintain their functional integrity. If so, where these regions of the genome have been duplicated, there may be an evolutionary advantage in maintaining the duplications, either through necessity developed after the duplication, as implied by subfunctionalisation, or through 'buffering by dosage' against deleterious mutations. The converse argument might be that within cold-spots more rapid sequence evolution might be driven by the development of rice-specific adaptation (and by extension to other genomes, ryegrass, maize or barley-specific adaptation). Exceptions to this general trend are the duplicated terminal segments on Pm11 and Pm12, which are relative cold-spots. However, as these are more recent duplications (c. 5 million years ago) and of a limited scale (*i.e*. not originating from effective polyploidisation) the situation may not be fully analogous [36,51]. Leaving Pm11 and Pm12 aside, over an evolutionary timescale, processes active in the maintenance of paralogous genes would be expected to work at the level of the gene and not on a region of a chromosome, as suggested by the correlations and heat-maps presented here. Indeed, within the duplicated segments there is also an indication that the actual FAexpTRL by which the segmental duplications are recognised show a greater degree of sequence conservation, in terms of numbers of alignments with the different plant databases, than the FAexpTRL for which no duplication is recognised (data not shown). However, this trend is not reflected in relatively higher MegaBLAST scores for these sequences and is also complicated by uncertainty in defining the precise starts and ends of duplicated segments and the presence of, apparently, multiply duplicated segments. It is also possible that, if one of the main processes driving the retention of paralogs is their potential significance in the maintenance of dosage sensitive gene networks [47], then what is being reflected in the heat maps is the physical association in chromosomal regions of genes interrelated through their metabolic functions.

The complete sequence of the rice genome and the rapid development in the complete sequencing of the maize and *Brachypodium distachyon *genomes are new resources for developing evolutionary models for monocots. Additionally, they represent invaluable tools for increasing our understanding of the structure and function of the larger grass and cereal genomes of the Poaceae. Technologies are still in development that will enable the efficient and accurate complete sequencing, reconstruction and annotation of these large grass and cereal genomes. Until these are established, comparative genome analysis will remain the underpinning, unifying approach to understanding the evolutionary (including the process of domestication) mechanisms that have led to the modern day plant genome. The present study has developed an approach to identifying and illustrating similarities and differences between coding sequences related to the physical position rice of putative orthologous gene models. It has thus contributed to the developing understanding of the internal structure of plant genomes and their interrelationships.

## Conclusion

Comparisons of patterns of rice pseudomolecule-anchored sequence alignments between rice FAexpTRL and transcript-assembly and methylation-filtered databases from other plant species indicated that the relative numbers of alignments were not-randomly distributed throughout the rice genome; rank correlations between relative physical position in rice and % aligned sequences were positive and significant for all the plant species (Table 1). When the relationships were illustrated using heat-maps of colour coded moving average windows of % alignments/queried database/100 FAexpTRL (Figure 3) it became apparent that particular pseudomolecules and regions within pseudomolecules contained FAexpTRL which were relatively more (eg. Pm3) or less (eg. Pm11 and Pm12) conserved than other pseudomolecules. The observed patterns were consistent (particularly within the monocots) across comparisons with different plant species. Furthermore, when a comparison of the relative positions of ancestrally segmentally duplicated regions of the rice genome was made with the relative numbers of percentage alignments with the test databases, the two were found to be co-incident in terms of both positive, significant correlations (Table 3) and heat-maps (Figure 4). Analysis of the nature of the genes present in hot-spots and cold-spots, inferred from the published annotations, indicated that Pm3 and Pm11 were enriched for FAexpTRL with unique annotations and those contained within large gene families, respectively. Additionally, members of certain of the large gene families, particularly F-box, LRR and NB-ARC domain containing proteins were under-represented in the hot-spots and over-represented in cold-spots (Table 6). Maximal expression patterns of *Arabidopsis *'orthologues' of FAexpTRL present in hot-spots showed a slight enrichment for tissues directly associated with meristematic cell division (particularly lateral root cap and callus; Table 4) and for growth stages associated with germination and floral initiation (Table 5). While, the evidence from these results alone is insufficient to conclude that hot-spots and cold-spots reflect the presence of different gene types and processes, for instance rice-specific or non-specific gene activities and networks, further investigation is warranted. As comprehensive genomic and transcriptomic resources become available for more plant species, these investigations will be facilitated.

## Methods

### Plant DNA databases

Details and sources of the databases developed for *Oryza sativa *(rice), *Lolium perenne *(perennial ryegrass), *Zea mays *(maize), *Hordeum vulgare *(barley), *Glycine max *(soybean) and *Arabidopsis thaliana *(thale cress) and their abbreviations used in this study are described in Table 7.

**Table 7:** DNA databases

**Species**	Sequence type	Abbreviation	No. sequences	Total base pairs	Average sequence length	Source
*Oryza sativa*	CDS	Os_CD	62827	85784595	1365	TIGR
*Lolium perenne*	MF	Lp_MF	471749	236911323	502	ViaLactia
*Zea mays*	MF	Zm_MF	450197	338653263	752	NCBI
*Zea mays*	TA	Zm_TA	169087	112449533	665	TIGR
*Hordeum_vulgare*	TA	Hv_TA	123351	83655311	678	TIGR
*Glycine max*	TA	Gm_TA	114693	65479263	571	TIGR
*Arabidopsis_thaliana*	TA	At_TA	148368	92081906	621	TIGR
*Arabidopsis_thaliana*	CDS	At_CD	30690	38043579	1240	TAIR

MF = methylation filtered

CDS = predicted complete coding sequences

TA = transcript assemblies

ViaLactia = ViaLactia Biosciences [60].

NCBI = National Center for Biotechnology Information [61]. Download command line = '(txid4577 [ORGN] AND Quackenbush [AUTH] AND "methylation" [ALL])' obtained from Gramene [9].

TIGR = The Institute for Genome Research [52, 62].

TAIR = The Arabidopsis Information Resource [11].

For different parts of the analysis, the rice CDS database (Os_CD) was subdivided into groups based upon the annotation assigned to the individual TIGR rice loci (TRL). Annotations [originally obtained from the file 'all.TU_model.brief_info.4', detailed in Additional file 6] are available from the TIGR FTP directory [52]. Subdivisions were 1) transposon or retrotransposon related TRL; 2) Hypothetical proteins; 3) Expressed proteins; 4) All functionally annotated (FA) TRL; 5) Expressed functionally annotated (FAexp) TRL [see Additional file 2 Table 1 and Additional file 6].

Additional subdivisions of the FAexpTRL Os_CD sequences were made based on their relative physical order in the individual rice pseudomolecules; each division consisted of a 'linear' 10% of the FAexpTRL assigned to each pseudomolecule (the final division for each pseudomolecule consisted of 10% of the FAexpTRL +/- the remainder) [see Additional file 2 Table 2].

The relative positions of FAexpTRL identified as being segmentally duplicated within the rice genome were obtained from TIGR, rice genome annotation, 500 kb rice genome semental duplication database [38].

### Sequence alignments

Sequence alignments were performed using standalone MegaBLAST [53] obtained from NCBI, BLAST FTP site [54] with default settings except for a window size of 16 (-W 16), maximum expectation value of 1 × 10^-10 ^(-e 1e-010) and alignment output (-D 3). Six different searches were performed, each one consisting of one of the 6 transcript assembly (TA) or methylation-filtered (MF) databases described in Table 1 (collectively, the test databases) queried with the Os_CD database.

### MegaBLAST output analysis

From each MegaBLAST search the number of TRL aligned to sequences from each of the other databases above the cut-off thresholds was recorded along with the associated scores. Where multiple significant alignments/TRL were identified within one of the test databases, the alignment with the highest score was used.

#### Linear 10% assignments

For each linear 10% pseudomolecule division, the number of significant alignments from each test database was calculated as a percentage of the total number of FAexpTRL in the Os_CD database. Additionally, the proportion of segmentally duplicated FAexpTRL in relation to the total number of FAexpTRL per 10% pseudomolecule division was calculated. Spearman rank correlation coefficients and associated probabilities were generated using GenStat for Windows^® ^(8.1) [55].

#### Moving windows (MWs)

MWs for the individual test databases were calculated on the basis of: 1) the % of FAexpTRL aligned with the test database above the cut-off thresholds per 100 consecutive FAexpTRL on each rice pseudomolecule, 2) the average score of the alignments with the test database above the cut-off thresholds in each group of 100 consecutive FAexp TRL on each rice pseudomolecule. Additionally MWs were calculated for 1) average alignment data from the 4 monocot databases; each FAexpTRL was assigned a value of 0, 0.25, 0.5, 0.75 or 1 based on the proportion of the monocot databases with which it was aligned. MWs were then calculated for the average alignment score/100 consecutive FAexpTRL; 2) the relative proportion of segmentally duplicated FAexpTRL thresholds in each group of 100 consecutive FAexpTRL on each rice pseudomolecule and 3) average gene family size in each group of 100 consecutive FAexpTRL on each rice pseudomolecule [see Additional files 1 and 7].

#### Data displays

For Figure 2, each TRL is represented by a colour coded horizontal bar. The horizontal bars are arranged, equidistantly, in a linear vertical order which represents their physical order on their respective rice pseudomolecules but does not directly reflect their physical or genetic distance from each other. For Figures 3, 4 and Additional file 4, the linear order of the MWs reflects the linear physical order of the FAexpTRL on each rice pseudomolecule. The data are displayed in the form of a 'heat-map' consisting of 10 colours (blue<red) with each colour representing 10% (or as close to as the data allowed) of the MWs ranked according to either increasing % alignments, increasing average scores, increasing number of segmentally duplicated FAexpTRL or decreasing average gene family size [see Additional file 2 Table 3 for key].

#### Arabidopsis expression profiles

FAexpTRL present in the highest 10% average MWs (red colour code) [see Additional file 2 Table 3] which had significant alignments with all the monocot test databases were queried against the At_CD database using MegaBLAST with the parameters as described previously. The expression profiles of significant *Arabidopsis *gene model alignments from the At_CD database [see Additional file 6] were obtained through the Genevestigator^® ^Meta-Analyzer database [56,57] for both growth stage and plant organs. For each gene model, the growth stage and the plant organ which showed maximal expression were ascertained and maximal expression profiles for growth stage and plant organs relative to the *Arabidopsis *gene models were constructed. The profiles developed from the FAexpTRL identified in the highest 10% MWs were compared with profiles developed from a random sample of 1599 FAexp *Arabidopsis *gene models.

## Abbreviations

TRL = TIGR rice locus

FA = Functionally annotated

FAexp = Functionally annotated, expressed

MW = moving window

Pm1, 2 etc. = TIGR rice pseudomolecule 1, 2, etc.

## Authors' contributions

IA designed and executed the analysis, all authors contributed to data interpretation, manuscript preparation and read and approved the final version.

## Supplementary Material

Additional file 2 Contains supplementary tables: Additional file 2 Table 1 details the %MegaBLAST alignments between the different TRL categories in the Os_CD database and the other plant databases. Additional file 2 Table 2 details the % MegaBLAST alignments and average scores of linear 10% segments of FAexpTRL for each pseudomolecule for each plant database. Additional file 2 Table 3 details the relation between colour codes used in the MW displays and the distribution of percentage alignments, average scores, segmentally duplicated FAexpTRL and gene family sizes. Additional file 2 Table 4 details derived gene family sizes used in the calculation of MWs for gene family size. Click here for file

Additional file 3 Illustrates the %MegaBLAST alignments/rice pseudomolecule between FAexpTRL and the plant databases. Click here for file

Additional file 7 Lists moving window (MW) scores used to produce Figures 3, 4 and Additional file 4. Click here for file

Additional file 1 Supplementary methods describing the derivation of FAexp gene family sizes based upon identical annotations. Click here for file

Additional file 4 Illustrates colour coded MWs comparing %alignments between the Os_CD database and the test databases in relation to average gene family size. Click here for file

Additional file 6 Lists 1)TIGR rice loci model identifiers and annotation categories; 2) *Arabidopsis *gene models used in MegaBLAST analyses; 3) TIGR rice loci gene families and sizes based upon identical annotations. Click here for file

Additional file 5 Contains MegaBLAST scores for the test databases against Os_CD. Click here for file
