# Supplementary material for: Rice pseudomolecule-anchored cross-species DNA sequence alignments indicate regional genomic variation in expressed sequence conservation
Source: BMC Genomics. 2007 Aug 20;8:283. doi: 10.1186/1471-2164-8-283 (PMC2041955; doi:10.1186/1471-2164-8-283)
Supplement: Additional file 2 — Contains supplementary tables: Additional file 2 Table 1 details the %MegaBLAST alignments between the different TRL categories in the Os_CD database and the other plant databases. Additional file 2 Table 2 details the % MegaBLAST alignments and average scores of linear 10% segments of FAexpTRL for each pseudomolecule for each plant database. Additional file 2 Table 3 details the relation between colour codes used in the MW displays and the distribution of percentage alignments, average scores, segmentally duplicated FAexpTRL and gene family sizes. Additional file 2 Table 4 details derived gene family sizes used in the calculation of MWs for gene family size. [file 1471-2164-8-283-S2.doc]

| Annotation | Test | Rice Pseudomolecule | | | | | | | | | | | | |
| --- | --- | --- | --- | --- | --- | --- | --- | --- | --- | --- | --- | --- | --- | --- |
| Category | database | All | 1 | 2 | 3 | 4 | 5 | 6 | 7 | 8 | 9 | 10 | 11 | 12 |
| All TIGR rice loci (TRL) |  | **55890** | **6594** | **5341** | **5558** | **5340** | **4598** | **4737** | **4488** | **4191** | **3391** | **3439** | **4198** | **4015** |
|  | Lp_MF | 37 | 43 | 41 | 43 | 36 | 36 | 36 | 37 | 34 | 36 | 35 | 29 | 30 |
|  | Zm_MF | 43 | 50 | 50 | 54 | 41 | 44 | 42 | 41 | 39 | 40 | 37 | 32 | 34 |
|  | Zm_TA | 45 | 53 | 52 | 56 | 43 | 46 | 43 | 43 | 41 | 42 | 39 | 32 | 36 |
|  | Hv_TA | 42 | 49 | 48 | 52 | 40 | 43 | 40 | 40 | 38 | 39 | 38 | 29 | 33 |
|  | Gm_TA | 10 | 12 | 13 | 14 | 8 | 11 | 9 | 9 | 9 | 9 | 8 | 6 | 7 |
|  | At_TA | 7 | 9 | 10 | 11 | 6 | 8 | 7 | 7 | 7 | 6 | 6 | 4 | 6 |
| FAexp |  | **17108** | **2494** | **1994** | **2228** | **1521** | **1370** | **1402** | **1321** | **1127** | **933** | **911** | **912** | **895** |
|  | Lp_MF | 67 | 71 | 67 | 70 | 67 | 66 | 67 | 67 | 63 | 67 | 65 | 58 | 58 |
|  | Zm_MF | 80 | 83 | 82 | 86 | 79 | 82 | 78 | 80 | 78 | 80 | 74 | 68 | 73 |
|  | Zm_TA | 84 | 87 | 86 | 91 | 83 | 87 | 82 | 84 | 82 | 84 | 79 | 71 | 77 |
|  | Hv_TA | 83 | 85 | 84 | 88 | 81 | 85 | 82 | 82 | 80 | 83 | 78 | 71 | 77 |
|  | Gm_TA | 28 | 28 | 31 | 31 | 23 | 30 | 26 | 27 | 27 | 28 | 24 | 21 | 26 |
|  | At_TA | 21 | 21 | 24 | 25 | 18 | 22 | 21 | 22 | 21 | 20 | 18 | 16 | 20 |
| FA |  | **21385** | **2955** | **2383** | **2565** | **1913** | **1660** | **1795** | **1685** | **1455** | **1172** | **1223** | **1367** | **1212** |
|  | Lp_MF | 63 | 67 | 64 | 68 | 62 | 62 | 63 | 62 | 59 | 65 | 61 | 52 | 53 |
|  | Zm_MF | 73 | 78 | 78 | 83 | 72 | 77 | 73 | 71 | 70 | 74 | 66 | 57 | 65 |
|  | Zm_TA | 76 | 81 | 81 | 85 | 73 | 80 | 74 | 74 | 72 | 76 | 69 | 57 | 66 |
|  | Hv_TA | 74 | 79 | 78 | 82 | 72 | 78 | 73 | 73 | 70 | 76 | 69 | 58 | 65 |
|  | Gm_TA | 26 | 28 | 30 | 31 | 23 | 30 | 25 | 25 | 25 | 27 | 22 | 17 | 25 |
|  | At_TA | 18 | 18 | 21 | 23 | 16 | 19 | 18 | 18 | 17 | 17 | 15 | 11 | 16 |
| Expressed proteins |  | **6911** | **972** | **775** | **850** | **582** | **590** | **536** | **524** | **479** | **381** | **352** | **445** | **425** |
|  | Lp_MF | 36 | 39 | 36 | 41 | 37 | 38 | 34 | 37 | 31 | 36 | 34 | 30 | 28 |
|  | Zm_MF | 48 | 52 | 53 | 57 | 48 | 49 | 46 | 43 | 42 | 46 | 44 | 35 | 40 |
|  | Zm_TA | 51 | 54 | 56 | 61 | 51 | 53 | 49 | 48 | 43 | 49 | 46 | 37 | 44 |
|  | Hv_TA | 48 | 52 | 53 | 56 | 48 | 51 | 48 | 44 | 40 | 48 | 44 | 33 | 42 |
|  | Gm_TA | 6 | 6 | 7 | 6 | 7 | 7 | 7 | 6 | 4 | 7 | 5 | 4 | 6 |
|  | At_TA | 4 | 4 | 4 | 4 | 3 | 5 | 3 | 3 | 4 | 3 | 3 | 3 | 4 |
| Hypothetical proteins |  | **14337** | **1504** | **1212** | **1234** | **1318** | **1155** | **1243** | **1201** | **1166** | **929** | **955** | **1294** | **1126** |
|  | Lp_MF | 8 | 9 | 8 | 8 | 9 | 9 | 7 | 9 | 9 | 7 | 8 | 6 | 7 |
|  | Zm_MF | 9 | 10 | 10 | 12 | 11 | 12 | 10 | 8 | 9 | 8 | 8 | 8 | 9 |
|  | Zm_TA | 10 | 9 | 9 | 10 | 11 | 12 | 11 | 9 | 11 | 10 | 8 | 8 | 10 |
|  | Hv_TA | 7 | 8 | 7 | 7 | 7 | 9 | 7 | 6 | 8 | 7 | 8 | 5 | 6 |
|  | Gm_TA | <0.05 | 1 | <0.05 | <0.05 | <0.05 | <0.05 | <0.05 | <0.05 | 1 | 1 | <0.05 | <0.05 | <0.05 |
|  | At_TA | <0.05 | <0.05 | <0.05 | <0.05 | <0.05 | <0.05 | <0.05 | <0.05 | <0.05 | 0 | <0.05 | <0.05 | 1 |
| Retro/ transposon |  | **13257** | **1163** | **971** | **909** | **1527** | **1193** | **1163** | **1078** | **1091** | **909** | **909** | **1092** | **1252** |
|  | Lp_MF | 28 | 27 | 28 | 24 | 27 | 27 | 26 | 30 | 30 | 28 | 28 | 28 | 28 |
|  | Zm_MF | 28 | 29 | 28 | 30 | 28 | 29 | 26 | 32 | 28 | 26 | 27 | 27 | 26 |
|  | Zm_TA | 29 | 34 | 32 | 29 | 30 | 29 | 27 | 30 | 32 | 26 | 27 | 28 | 28 |
|  | Hv_TA | 23 | 24 | 23 | 24 | 24 | 24 | 22 | 24 | 26 | 22 | 25 | 20 | 22 |
|  | Gm_TA | 1 | 1 | 1 | 2 | 1 | 1 | <0.05 | 1 | 1 | 1 | 1 | 1 | 1 |
|  | At_TA | <0.05 | 1 | <0.05 | 1 | <0.05 | <0.05 | <0.05 | <0.05 | <0.05 | 0 | <0.05 | <0.05 | <0.05 |

Additional file 2 Table 1. Numbers of significant alignments between sequences from different annotation categories from the Os_CD database and the 6 plant test databases. Results (% of the total in each category) are expressed in terms of the whole genome (All) and the individual rice pseudomolecules (1-12).

TRL = TIGR rice locus

FA = Functionally annotated

FAexp = Functionally annotated, expressed

Bold type indicates the total number of TRL in each individual annotation category.

Additional file 2 Table 2. Percentage MegaBLAST alignments and means scores for each linear 10% of FAexpTRL from each pseudomolecule with each test database

| **Os_CD** | | | |  | **Lp_MF** | |  | **Zm_MF** | |  | **Zm_TA** | |  | **Hv_TA** | |  | **Gm_TA** | |  | **At_TA** | |
| --- | --- | --- | --- | --- | --- | --- | --- | --- | --- | --- | --- | --- | --- | --- | --- | --- | --- | --- | --- | --- | --- |
| **Pseudomoleule segment** | **FAexp TRL (A)** | **Segmentally duplicated FAexp TRL (B)** | **A/B^1^** |  | **%** | **Mean score** |  | **%** | **Mean score** |  | **%** | **Mean score** |  | **%** | **Mean score** |  | **%** | **Mean score** |  | **%** | **Mean score** |
| **1.1** | **249** | **27** | **0.11** |  | **68.7** | **209.0** |  | **81.9** | **390.1** |  | **76.7** | **244.8** |  | **79.9** | **434.5** |  | **24.9** | **135.7** |  | **15.7** | **120.4** |
| **1.2** | **249** | **77** | **0.31** |  | **74.3** | **210.0** |  | **85.9** | **456.4** |  | **85.1** | **240.1** |  | **87.1** | **473.8** |  | **26.5** | **134.1** |  | **19.3** | **144.5** |
| **1.3** | **249** | **30** | **0.12** |  | **67.9** | **207.0** |  | **82.7** | **495.9** |  | **82.7** | **264.6** |  | **87.6** | **512.6** |  | **32.9** | **177.8** |  | **24.5** | **164.6** |
| **1.4** | **249** | **15** | **0.06** |  | **63.5** | **185.0** |  | **80.3** | **483.7** |  | **73.1** | **243.7** |  | **81.9** | **526.1** |  | **22.1** | **157.2** |  | **16.1** | **157.5** |
| **1.5** | **249** | **48** | **0.19** |  | **71.9** | **205.0** |  | **83.9** | **489.9** |  | **81.9** | **281.6** |  | **85.1** | **519.1** |  | **26.5** | **162.1** |  | **22.1** | **145.7** |
| **1.6** | **249** | **94** | **0.38** |  | **74.7** | **229.0** |  | **86.7** | **480.2** |  | **83.9** | **258.0** |  | **89.2** | **492.5** |  | **31.3** | **169.0** |  | **24.9** | **172.2** |
| **1.7** | **249** | **75** | **0.30** |  | **74.7** | **224.0** |  | **88.0** | **488.0** |  | **82.3** | **277.3** |  | **88.4** | **511.9** |  | **30.5** | **161.3** |  | **22.1** | **163.8** |
| **1.8** | **249** | **123** | **0.49** |  | **73.5** | **247.0** |  | **88.4** | **484.0** |  | **88.8** | **312.5** |  | **92.4** | **540.4** |  | **32.5** | **160.0** |  | **23.3** | **150.2** |
| **1.9** | **249** | **70** | **0.28** |  | **70.3** | **221.0** |  | **81.5** | **515.1** |  | **87.6** | **264.8** |  | **90.0** | **523.1** |  | **27.3** | **139.7** |  | **21.3** | **124.2** |
| **1.10** | **253** | **54** | **0.21** |  | **72.3** | **201.0** |  | **90.1** | **412.7** |  | **84.2** | **241.8** |  | **88.1** | **462.8** |  | **27.7** | **139.5** |  | **20.6** | **138.7** |
|  |  |  |  |  |  |  |  |  |  |  |  |  |  |  |  |  |  |  |  |  |  |
| **2.1** | **199** | **23** | **0.12** |  | **63.8** | **199.0** |  | **85.9** | **528.7** |  | **82.4** | **253.1** |  | **90.5** | **567.5** |  | **33.7** | **167.2** |  | **30.7** | **159.5** |
| **2.2** | **199** | **74** | **0.37** |  | **71.4** | **200.0** |  | **89.4** | **496.9** |  | **78.4** | **236.3** |  | **85.4** | **528.7** |  | **28.6** | **166.4** |  | **26.1** | **168.6** |
| **2.3** | **199** | **67** | **0.34** |  | **64.8** | **188.0** |  | **80.9** | **473.5** |  | **79.4** | **234.5** |  | **80.9** | **540.5** |  | **28.6** | **162.1** |  | **22.6** | **174.9** |
| **2.4** | **199** | **0** | **0.00** |  | **55.8** | **216.0** |  | **73.4** | **415.7** |  | **67.3** | **222.2** |  | **80.4** | **458.9** |  | **25.1** | **169.5** |  | **20.1** | **163.9** |
| **2.5** | **199** | **67** | **0.34** |  | **62.8** | **194.0** |  | **81.9** | **468.7** |  | **77.9** | **220.0** |  | **83.4** | **508.7** |  | **29.6** | **159.0** |  | **22.6** | **158.0** |
| **2.6** | **199** | **76** | **0.38** |  | **64.3** | **190.0** |  | **81.4** | **498.7** |  | **82.4** | **280.2** |  | **83.9** | **542.9** |  | **29.6** | **152.5** |  | **24.1** | **143.4** |
| **2.7** | **199** | **120** | **0.60** |  | **77.4** | **228.0** |  | **86.9** | **447.6** |  | **89.9** | **276.8** |  | **89.4** | **477.5** |  | **37.2** | **123.0** |  | **22.1** | **134.8** |
| **2.8** | **199** | **73** | **0.37** |  | **72.9** | **237.0** |  | **88.4** | **526.3** |  | **85.9** | **308.7** |  | **91.5** | **581.8** |  | **29.6** | **139.7** |  | **23.6** | **139.8** |
| **2.9** | **199** | **83** | **0.42** |  | **71.9** | **227.0** |  | **87.9** | **503.3** |  | **92.0** | **312.8** |  | **93.5** | **560.0** |  | **29.6** | **133.6** |  | **20.6** | **136.2** |
| **2.10** | **203** | **25** | **0.12** |  | **62.1** | **193.0** |  | **86.7** | **512.6** |  | **83.7** | **261.8** |  | **85.2** | **585.5** |  | **34.0** | **152.8** |  | **23.6** | **143.9** |
|  |  |  |  |  |  |  |  |  |  |  |  |  |  |  |  |  |  |  |  |  |  |
| **3.1** | **223** | **84** | **0.38** |  | **67.7** | **246.0** |  | **88.8** | **490.1** |  | **87.0** | **287.8** |  | **91.9** | **526.7** |  | **26.0** | **159.8** |  | **24.7** | **133.9** |
| **3.2** | **223** | **73** | **0.33** |  | **73.1** | **215.0** |  | **90.1** | **490.8** |  | **91.9** | **286.8** |  | **93.7** | **531.6** |  | **28.3** | **193.2** |  | **26.5** | **200.1** |
| **3.3** | **223** | **21** | **0.09** |  | **76.2** | **264.0** |  | **88.3** | **561.0** |  | **88.8** | **326.1** |  | **92.4** | **619.3** |  | **27.8** | **165.1** |  | **19.3** | **152.4** |
| **3.4** | **223** | **55** | **0.25** |  | **72.6** | **244.0** |  | **93.3** | **591.2** |  | **87.4** | **344.9** |  | **95.5** | **618.9** |  | **36.8** | **142.5** |  | **31.4** | **137.2** |
| **3.5** | **223** | **91** | **0.41** |  | **71.7** | **213.0** |  | **87.4** | **482.5** |  | **81.6** | **254.4** |  | **90.6** | **529.5** |  | **30.0** | **143.3** |  | **23.3** | **134.4** |
| **3.6** | **223** | **40** | **0.18** |  | **65.0** | **233.0** |  | **84.3** | **440.3** |  | **82.1** | **270.0** |  | **87.0** | **489.1** |  | **30.9** | **171.0** |  | **25.6** | **147.9** |
| **3.7** | **223** | **55** | **0.25** |  | **66.8** | **205.0** |  | **82.1** | **475.3** |  | **79.8** | **259.2** |  | **86.1** | **536.4** |  | **30.5** | **152.9** |  | **22.4** | **167.6** |
| **3.8** | **223** | **3** | **0.01** |  | **70.9** | **233.0** |  | **88.3** | **617.7** |  | **88.3** | **299.1** |  | **91.0** | **631.8** |  | **32.7** | **160.8** |  | **28.7** | **165.5** |
| **3.9** | **223** | **79** | **0.35** |  | **70.9** | **218.0** |  | **93.3** | **483.1** |  | **90.1** | **274.5** |  | **90.6** | **529.7** |  | **32.7** | **178.0** |  | **28.3** | **145.8** |
| **3.10** | **221** | **17** | **0.08** |  | **67.9** | **186.0** |  | **87.8** | **503.3** |  | **87.3** | **235.3** |  | **87.8** | **523.1** |  | **30.3** | **163.7** |  | **24.9** | **165.2** |
|  |  |  |  |  |  |  |  |  |  |  |  |  |  |  |  |  |  |  |  |  |  |
| **4.1** | **152** | **0** | **0.00** |  | **56.6** | **175.0** |  | **72.4** | **384.7** |  | **63.2** | **236.4** |  | **67.1** | **398.8** |  | **13.8** | **154.3** |  | **12.5** | **152.3** |
| **4.2** | **152** | **12** | **0.08** |  | **55.9** | **226.0** |  | **67.8** | **403.0** |  | **58.6** | **277.0** |  | **68.4** | **421.5** |  | **18.4** | **198.7** |  | **17.8** | **207.5** |
| **4.3** | **152** | **48** | **0.32** |  | **56.6** | **199.0** |  | **80.9** | **481.9** |  | **71.1** | **256.3** |  | **80.3** | **516.7** |  | **24.3** | **159.6** |  | **21.1** | **151.1** |
| **4.4** | **152** | **44** | **0.29** |  | **78.3** | **220.0** |  | **85.5** | **514.2** |  | **88.2** | **285.1** |  | **88.8** | **511.5** |  | **25.7** | **139.2** |  | **21.1** | **137.3** |
| **4.5** | **152** | **34** | **0.22** |  | **71.7** | **232.0** |  | **79.6** | **454.7** |  | **84.9** | **270.3** |  | **82.9** | **477.8** |  | **24.3** | **143.8** |  | **18.4** | **165.3** |
| **4.6** | **152** | **73** | **0.48** |  | **73.0** | **221.0** |  | **86.2** | **486.9** |  | **88.8** | **288.7** |  | **94.7** | **514.4** |  | **25.0** | **142.6** |  | **20.4** | **125.1** |
| **4.7** | **152** | **75** | **0.49** |  | **77.0** | **226.0** |  | **83.6** | **415.0** |  | **90.1** | **322.8** |  | **87.5** | **484.8** |  | **22.4** | **135.2** |  | **13.2** | **130.0** |
| **4.8** | **152** | **41** | **0.27** |  | **68.4** | **233.0** |  | **84.9** | **444.7** |  | **77.6** | **262.8** |  | **84.2** | **468.7** |  | **22.4** | **146.1** |  | **17.1** | **135.4** |
| **4.9** | **152** | **17** | **0.11** |  | **69.1** | **209.0** |  | **84.2** | **535.6** |  | **84.2** | **251.4** |  | **84.9** | **557.1** |  | **33.6** | **137.2** |  | **21.7** | **145.3** |
| **4.10** | **153** | **27** | **0.18** |  | **66.7** | **172.0** |  | **83.7** | **411.7** |  | **85.6** | **240.3** |  | **90.8** | **462.9** |  | **24.8** | **133.7** |  | **20.9** | **146.1** |
|  |  |  |  |  |  |  |  |  |  |  |  |  |  |  |  |  |  |  |  |  |  |
| **5.1** | **137** | **46** | **0.34** |  | **67.2** | **193.0** |  | **83.9** | **471.8** |  | **81.0** | **240.1** |  | **89.1** | **444.8** |  | **29.9** | **167.3** |  | **21.2** | **167.7** |
| **5.2** | **137** | **48** | **0.35** |  | **63.5** | **187.0** |  | **86.9** | **591.7** |  | **73.0** | **246.8** |  | **83.9** | **635.9** |  | **31.4** | **170.3** |  | **24.8** | **157.7** |
| **5.3** | **137** | **47** | **0.34** |  | **59.9** | **206.0** |  | **82.5** | **441.4** |  | **77.4** | **216.8** |  | **81.8** | **448.2** |  | **26.3** | **147.6** |  | **16.8** | **133.7** |
| **5.4** | **137** | **7** | **0.05** |  | **56.9** | **243.0** |  | **73.0** | **500.5** |  | **70.8** | **259.4** |  | **78.8** | **509.4** |  | **26.3** | **157.4** |  | **18.2** | **150.3** |
| **5.5** | **137** | **34** | **0.25** |  | **59.1** | **209.0** |  | **86.9** | **505.8** |  | **79.6** | **233.3** |  | **84.7** | **474.0** |  | **27.7** | **154.5** |  | **21.2** | **166.0** |
| **5.6** | **137** | **60** | **0.44** |  | **75.2** | **251.0** |  | **89.8** | **542.3** |  | **90.5** | **336.2** |  | **88.3** | **551.1** |  | **25.5** | **161.6** |  | **24.8** | **135.7** |
| **5.7** | **137** | **89** | **0.65** |  | **67.9** | **263.0** |  | **87.6** | **481.2** |  | **84.7** | **299.0** |  | **92.0** | **520.7** |  | **32.8** | **167.1** |  | **20.4** | **166.5** |
| **5.8** | **137** | **62** | **0.45** |  | **69.3** | **210.0** |  | **92.0** | **508.6** |  | **89.1** | **255.1** |  | **92.0** | **529.0** |  | **33.6** | **152.7** |  | **24.1** | **139.8** |
| **5.9** | **137** | **77** | **0.56** |  | **68.6** | **200.0** |  | **88.3** | **446.1** |  | **86.9** | **230.7** |  | **92.7** | **497.1** |  | **34.3** | **142.8** |  | **20.4** | **166.4** |
| **5.10** | **137** | **65** | **0.47** |  | **72.3** | **180.0** |  | **81.8** | **484.2** |  | **89.1** | **253.7** |  | **89.8** | **505.7** |  | **33.6** | **155.2** |  | **24.8** | **164.5** |
|  |  |  |  |  |  |  |  |  |  |  |  |  |  |  |  |  |  |  |  |  |  |
| **6.1** | **140** | **5** | **0.04** |  | **62.1** | **206.0** |  | **82.1** | **524.6** |  | **83.6** | **278.0** |  | **91.4** | **524.3** |  | **30.7** | **156.3** |  | **27.9** | **144.7** |
| **6.2** | **140** | **7** | **0.05** |  | **66.4** | **213.0** |  | **77.1** | **474.5** |  | **80.0** | **229.5** |  | **82.1** | **512.1** |  | **28.6** | **177.7** |  | **21.4** | **155.2** |
| **6.3** | **140** | **44** | **0.31** |  | **74.3** | **202.0** |  | **82.9** | **468.5** |  | **79.3** | **271.3** |  | **85.7** | **508.1** |  | **18.6** | **185.0** |  | **18.6** | **165.3** |
| **6.4** | **140** | **58** | **0.41** |  | **75.0** | **209.0** |  | **82.9** | **433.3** |  | **76.4** | **286.3** |  | **81.4** | **471.1** |  | **25.7** | **138.9** |  | **17.9** | **132.1** |
| **6.5** | **140** | **24** | **0.17** |  | **58.6** | **202.0** |  | **76.4** | **444.3** |  | **67.9** | **243.5** |  | **74.3** | **412.1** |  | **22.9** | **128.6** |  | **16.4** | **113.2** |
| **6.6** | **140** | **18** | **0.13** |  | **58.6** | **171.0** |  | **75.7** | **419.0** |  | **65.0** | **245.7** |  | **72.1** | **444.5** |  | **16.4** | **128.9** |  | **13.6** | **128.7** |
| **6.7** | **140** | **50** | **0.36** |  | **85.0** | **222.0** |  | **89.3** | **522.5** |  | **83.6** | **290.5** |  | **85.0** | **530.8** |  | **29.3** | **180.1** |  | **23.6** | **168.6** |
| **6.8** | **140** | **34** | **0.24** |  | **63.6** | **221.0** |  | **81.4** | **483.5** |  | **85.7** | **282.0** |  | **85.0** | **527.9** |  | **30.0** | **147.2** |  | **22.9** | **146.0** |
| **6.9** | **140** | **36** | **0.26** |  | **62.1** | **220.0** |  | **82.1** | **475.7** |  | **81.4** | **275.6** |  | **82.1** | **518.7** |  | **30.7** | **172.6** |  | **23.6** | **189.8** |
| **6.10** | **142** | **27** | **0.19** |  | **62.0** | **194.0** |  | **87.3** | **458.6** |  | **81.7** | **234.3** |  | **83.1** | **521.6** |  | **31.0** | **145.7** |  | **24.6** | **167.4** |
|  |  |  |  |  |  |  |  |  |  |  |  |  |  |  |  |  |  |  |  |  |  |
| **7.1** | **132** | **26** | **0.20** |  | **68.2** | **176.0** |  | **83.3** | **389.1** |  | **78.0** | **198.6** |  | **78.0** | **412.2** |  | **21.2** | **193.5** |  | **18.2** | **157.6** |
| **7.2** | **132** | **43** | **0.33** |  | **70.5** | **203.0** |  | **87.1** | **482.1** |  | **85.6** | **232.4** |  | **88.6** | **508.7** |  | **29.5** | **137.8** |  | **21.2** | **142.3** |
| **7.3** | **132** | **32** | **0.24** |  | **56.1** | **191.0** |  | **68.2** | **459.1** |  | **68.9** | **230.5** |  | **73.5** | **502.7** |  | **25.0** | **189.8** |  | **25.8** | **158.9** |
| **7.4** | **132** | **0** | **0.00** |  | **59.8** | **167.0** |  | **77.3** | **455.9** |  | **72.7** | **217.2** |  | **78.8** | **498.8** |  | **28.8** | **129.1** |  | **22.0** | **140.5** |
| **7.5** | **132** | **0** | **0.00** |  | **56.8** | **195.0** |  | **81.1** | **458.4** |  | **78.8** | **232.5** |  | **86.4** | **500.1** |  | **31.1** | **129.5** |  | **17.4** | **152.8** |
| **7.6** | **132** | **0** | **0.00** |  | **70.5** | **209.0** |  | **76.5** | **405.1** |  | **72.7** | **272.5** |  | **77.3** | **422.1** |  | **22.7** | **166.5** |  | **20.5** | **150.6** |
| **7.7** | **132** | **0** | **0.00** |  | **74.2** | **223.0** |  | **90.2** | **521.3** |  | **84.1** | **327.6** |  | **90.9** | **529.5** |  | **25.0** | **186.5** |  | **23.5** | **158.1** |
| **7.8** | **132** | **46** | **0.35** |  | **66.7** | **222.0** |  | **87.9** | **495.4** |  | **82.6** | **253.4** |  | **87.9** | **495.2** |  | **32.6** | **180.6** |  | **22.0** | **153.9** |
| **7.9** | **132** | **32** | **0.24** |  | **74.2** | **190.0** |  | **88.6** | **466.5** |  | **85.6** | **239.2** |  | **88.6** | **471.0** |  | **23.5** | **134.5** |  | **20.5** | **138.1** |
| **7.10** | **133** | **71** | **0.53** |  | **75.9** | **205.0** |  | **82.7** | **469.0** |  | **86.5** | **234.2** |  | **91.7** | **479.9** |  | **29.3** | **163.4** |  | **24.8** | **151.9** |
|  |  |  |  |  |  |  |  |  |  |  |  |  |  |  |  |  |  |  |  |  |  |
| **8.1** | **113** | **9** | **0.08** |  | **61.9** | **196.0** |  | **81.4** | **444.3** |  | **78.8** | **224.2** |  | **77.9** | **445.6** |  | **23.9** | **139.6** |  | **19.5** | **127.2** |
| **8.2** | **113** | **2** | **0.02** |  | **53.1** | **213.0** |  | **74.3** | **432.0** |  | **82.3** | **231.1** |  | **84.1** | **426.6** |  | **29.2** | **123.1** |  | **21.2** | **118.2** |
| **8.3** | **113** | **26** | **0.23** |  | **62.8** | **187.0** |  | **68.1** | **440.9** |  | **69.9** | **193.2** |  | **73.5** | **548.6** |  | **31.0** | **132.4** |  | **21.2** | **151.5** |
| **8.4** | **113** | **9** | **0.08** |  | **51.3** | **181.0** |  | **80.5** | **398.0** |  | **62.8** | **225.9** |  | **71.7** | **466.4** |  | **21.2** | **118.5** |  | **17.7** | **115.9** |
| **8.5** | **113** | **7** | **0.06** |  | **61.9** | **201.0** |  | **69.9** | **526.2** |  | **70.8** | **223.0** |  | **77.0** | **560.3** |  | **25.7** | **152.0** |  | **27.4** | **122.5** |
| **8.6** | **113** | **39** | **0.35** |  | **61.1** | **198.0** |  | **76.1** | **388.8** |  | **78.8** | **194.2** |  | **85.8** | **409.2** |  | **25.7** | **131.7** |  | **17.7** | **120.6** |
| **8.7** | **113** | **46** | **0.41** |  | **75.2** | **244.0** |  | **81.4** | **468.8** |  | **84.1** | **324.5** |  | **88.5** | **476.0** |  | **22.1** | **185.4** |  | **15.0** | **168.3** |
| **8.8** | **113** | **42** | **0.37** |  | **77.0** | **223.0** |  | **88.5** | **417.0** |  | **77.9** | **267.1** |  | **85.0** | **456.7** |  | **31.0** | **155.5** |  | **24.8** | **148.8** |
| **8.9** | **113** | **39** | **0.35** |  | **55.8** | **187.0** |  | **85.8** | **495.9** |  | **82.3** | **237.4** |  | **82.3** | **538.7** |  | **30.1** | **142.1** |  | **24.8** | **123.4** |
| **8.10** | **110** | **46** | **0.42** |  | **70.9** | **226.0** |  | **89.1** | **526.0** |  | **91.8** | **290.3** |  | **95.5** | **505.5** |  | **32.7** | **177.5** |  | **25.5** | **151.8** |
|  |  |  |  |  |  |  |  |  |  |  |  |  |  |  |  |  |  |  |  |  |  |
| **9.1** | **93** | **0** | **0.00** |  | **54.8** | **212.0** |  | **82.8** | **521.5** |  | **68.8** | **240.8** |  | **77.4** | **543.2** |  | **23.7** | **199.5** |  | **19.4** | **171.5** |
| **9.2** | **93** | **8** | **0.09** |  | **55.9** | **197.0** |  | **76.3** | **493.6** |  | **65.6** | **235.4** |  | **73.1** | **535.5** |  | **26.9** | **146.1** |  | **24.7** | **122.8** |
| **9.3** | **93** | **13** | **0.14** |  | **63.4** | **155.0** |  | **78.5** | **447.1** |  | **71.0** | **240.7** |  | **78.5** | **473.2** |  | **24.7** | **181.7** |  | **14.0** | **183.6** |
| **9.4** | **93** | **27** | **0.29** |  | **61.3** | **198.0** |  | **78.5** | **521.0** |  | **89.2** | **264.9** |  | **89.2** | **482.9** |  | **29.0** | **112.4** |  | **16.1** | **119.6** |
| **9.5** | **93** | **30** | **0.32** |  | **77.4** | **220.0** |  | **83.9** | **448.9** |  | **90.3** | **311.4** |  | **88.2** | **482.9** |  | **24.7** | **140.9** |  | **20.4** | **125.9** |
| **9.6** | **93** | **48** | **0.52** |  | **69.9** | **232.0** |  | **89.2** | **392.8** |  | **81.7** | **290.3** |  | **87.1** | **454.2** |  | **24.7** | **124.8** |  | **16.1** | **102.5** |
| **9.7** | **93** | **42** | **0.45** |  | **80.6** | **224.0** |  | **90.3** | **391.0** |  | **83.9** | **293.6** |  | **86.0** | **461.2** |  | **31.2** | **154.6** |  | **24.7** | **132.5** |
| **9.8** | **93** | **40** | **0.43** |  | **66.7** | **193.0** |  | **84.9** | **458.1** |  | **84.9** | **216.0** |  | **87.1** | **525.7** |  | **34.4** | **144.3** |  | **21.5** | **141.3** |
| **9.9** | **93** | **16** | **0.17** |  | **71.0** | **208.0** |  | **89.2** | **434.8** |  | **83.9** | **293.9** |  | **90.3** | **526.2** |  | **34.4** | **150.8** |  | **21.5** | **133.2** |
| **9.10** | **96** | **14** | **0.15** |  | **70.8** | **155.0** |  | **79.2** | **389.7** |  | **84.4** | **227.1** |  | **83.3** | **469.8** |  | **30.2** | **159.4** |  | **20.8** | **143.4** |
|  |  |  |  |  |  |  |  |  |  |  |  |  |  |  |  |  |  |  |  |  |  |
| **10.1** | **91** | **9** | **0.10** |  | **44.0** | **147.0** |  | **59.3** | **300.0** |  | **53.8** | **183.5** |  | **54.9** | **301.3** |  | **8.8** | **217.9** |  | **6.6** | **95.6** |
| **10.2** | **91** | **13** | **0.14** |  | **63.7** | **173.0** |  | **64.8** | **400.8** |  | **61.5** | **225.1** |  | **65.9** | **442.6** |  | **13.2** | **122.3** |  | **15.4** | **143.9** |
| **10.3** | **91** | **2** | **0.02** |  | **59.3** | **171.0** |  | **64.8** | **436.5** |  | **64.8** | **282.9** |  | **69.2** | **428.5** |  | **24.2** | **203.6** |  | **16.5** | **292.0** |
| **10.4** | **91** | **28** | **0.31** |  | **57.1** | **255.0** |  | **81.3** | **387.8** |  | **67.0** | **204.2** |  | **78.0** | **476.6** |  | **24.2** | **120.0** |  | **18.7** | **138.1** |
| **10.5** | **91** | **29** | **0.32** |  | **71.4** | **155.0** |  | **80.2** | **405.1** |  | **83.5** | **236.6** |  | **84.6** | **422.3** |  | **18.7** | **210.8** |  | **22.0** | **165.0** |
| **10.6** | **91** | **19** | **0.21** |  | **78.0** | **187.0** |  | **86.8** | **494.9** |  | **79.1** | **263.0** |  | **87.9** | **540.0** |  | **41.8** | **174.7** |  | **28.6** | **144.5** |
| **10.7** | **91** | **21** | **0.23** |  | **56.0** | **215.0** |  | **78.0** | **526.8** |  | **69.2** | **285.0** |  | **81.3** | **584.1** |  | **30.8** | **142.0** |  | **17.6** | **190.0** |
| **10.8** | **91** | **32** | **0.35** |  | **86.8** | **221.0** |  | **94.5** | **496.1** |  | **92.3** | **332.3** |  | **95.6** | **489.5** |  | **24.2** | **205.6** |  | **17.6** | **265.1** |
| **10.9** | **91** | **38** | **0.42** |  | **64.8** | **196.0** |  | **80.2** | **422.9** |  | **79.1** | **253.3** |  | **79.1** | **398.8** |  | **20.9** | **115.0** |  | **13.2** | **102.0** |
| **10.10** | **92** | **14** | **0.15** |  | **68.5** | **221.0** |  | **89.1** | **521.2** |  | **85.9** | **246.9** |  | **89.1** | **527.0** |  | **35.9** | **132.6** |  | **25.0** | **124.6** |
|  |  |  |  |  |  |  |  |  |  |  |  |  |  |  |  |  |  |  |  |  |  |
| **11.1** | **91** | **82** | **0.90** |  | **60.4** | **186.0** |  | **83.5** | **402.3** |  | **79.1** | **212.2** |  | **80.2** | **415.3** |  | **25.3** | **144.3** |  | **15.4** | **129.1** |
| **11.2** | **91** | **67** | **0.74** |  | **68.1** | **237.0** |  | **81.3** | **505.3** |  | **82.4** | **256.1** |  | **82.4** | **477.2** |  | **37.4** | **146.9** |  | **20.9** | **150.0** |
| **11.3** | **91** | **28** | **0.31** |  | **70.3** | **186.0** |  | **83.5** | **440.9** |  | **75.8** | **258.4** |  | **80.2** | **524.5** |  | **20.9** | **216.4** |  | **19.8** | **199.8** |
| **11.4** | **91** | **0** | **0.00** |  | **61.5** | **156.0** |  | **61.5** | **365.2** |  | **60.4** | **234.3** |  | **62.6** | **395.2** |  | **17.6** | **138.1** |  | **15.4** | **134.2** |
| **11.5** | **91** | **0** | **0.00** |  | **45.1** | **187.0** |  | **65.9** | **377.3** |  | **59.3** | **214.2** |  | **70.3** | **388.7** |  | **17.6** | **191.4** |  | **13.2** | **210.4** |
| **11.6** | **91** | **0** | **0.00** |  | **51.6** | **199.0** |  | **73.6** | **416.8** |  | **73.6** | **212.8** |  | **71.4** | **484.2** |  | **22.0** | **158.7** |  | **18.7** | **164.4** |
| **11.7** | **91** | **0** | **0.00** |  | **53.8** | **154.0** |  | **68.1** | **470.2** |  | **60.4** | **214.8** |  | **71.4** | **549.0** |  | **22.0** | **131.4** |  | **14.3** | **117.0** |
| **11.8** | **91** | **0** | **0.00** |  | **51.6** | **179.0** |  | **67.0** | **450.4** |  | **61.5** | **220.4** |  | **65.9** | **479.8** |  | **17.6** | **167.9** |  | **19.8** | **137.3** |
| **11.9** | **91** | **0** | **0.00** |  | **49.5** | **134.0** |  | **56.0** | **391.7** |  | **59.3** | **183.4** |  | **57.1** | **443.8** |  | **15.4** | **142.1** |  | **11.0** | **149.0** |
| **11.10** | **93** | **11** | **0.12** |  | **67.7** | **170.0** |  | **68.8** | **324.8** |  | **66.7** | **206.8** |  | **64.5** | **366.9** |  | **17.2** | **255.6** |  | **12.9** | **242.5** |
|  |  |  |  |  |  |  |  |  |  |  |  |  |  |  |  |  |  |  |  |  |  |
| **12.1** | **90** | **82** | **0.91** |  | **63.3** | **179.0** |  | **83.3** | **429.0** |  | **77.8** | **228.8** |  | **81.1** | **400.8** |  | **18.9** | **170.7** |  | **13.3** | **153.4** |
| **12.2** | **90** | **65** | **0.72** |  | **58.9** | **214.0** |  | **74.4** | **496.8** |  | **72.2** | **236.6** |  | **73.3** | **478.4** |  | **28.9** | **132.4** |  | **17.8** | **121.6** |
| **12.3** | **90** | **34** | **0.38** |  | **64.4** | **196.0** |  | **87.8** | **495.2** |  | **85.6** | **237.9** |  | **86.7** | **537.6** |  | **32.2** | **145.9** |  | **22.2** | **158.5** |
| **12.4** | **90** | **0** | **0.00** |  | **53.3** | **217.0** |  | **65.6** | **444.3** |  | **60.0** | **216.3** |  | **64.4** | **487.7** |  | **22.2** | **186.9** |  | **17.8** | **181.0** |
| **12.5** | **90** | **0** | **0.00** |  | **52.2** | **182.0** |  | **74.4** | **456.9** |  | **70.0** | **228.2** |  | **73.3** | **444.4** |  | **26.7** | **154.7** |  | **24.4** | **123.9** |
| **12.6** | **90** | **0** | **0.00** |  | **56.7** | **191.0** |  | **66.7** | **451.8** |  | **62.2** | **240.0** |  | **72.2** | **448.0** |  | **22.2** | **148.0** |  | **18.9** | **160.6** |
| **12.7** | **90** | **5** | **0.06** |  | **48.9** | **210.0** |  | **75.6** | **454.1** |  | **70.0** | **259.5** |  | **74.4** | **473.2** |  | **24.4** | **159.0** |  | **15.6** | **116.1** |
| **c12.8** | **90** | **17** | **0.19** |  | **62.2** | **190.0** |  | **78.9** | **457.7** |  | **73.3** | **242.5** |  | **76.7** | **524.4** |  | **25.6** | **132.0** |  | **25.6** | **129.1** |
| **c12.9** | **90** | **23** | **0.26** |  | **54.4** | **166.0** |  | **81.1** | **447.8** |  | **73.3** | **210.4** |  | **82.2** | **443.4** |  | **30.0** | **141.7** |  | **22.2** | **135.2** |
| **c12.10** | **85** | **27** | **0.32** |  | **69.4** | **217.0** |  | **83.5** | **525.6** |  | **82.4** | **256.3** |  | **83.5** | **534.3** |  | **30.6** | **143.8** |  | **27.1** | **169.4** |

Pseudomolecule segment = segment of each pseudomolecule (1-12) containing 10% of the FAexpTRL distributed on the basis of physical order

FAexpTRL = Number of functionally annotated, expressed TIGR rice loci in each pseudomolecule segment.

Segmentally duplicated FAexpTRL = number of segmentally duplicated FAexpTRL (see Materials and Methods) in each pseudomolecule segment.

^1^Represents a measure of the distribution of FAexpTRL identified as being segmentally duplicated between the linear 10% FAexpTRL groups for each pseudomolecule.

Additional file 2 Table 3. The relation between colour codes used in Figures 2,3 and Supplementary Figure 2, the distribution of % MegaBLAST alignments, average scores, segmentally duplicated FAexpTRL and gene family sizes for moving average windows of 100 FAexpTRL

| Database | MW type |  |  |  |  |  |  |  |  |  |  |
| --- | --- | --- | --- | --- | --- | --- | --- | --- | --- | --- | --- |
| Lp_MF | % alignments/MW | 42-55 | 56-59 | 60-63 | 64-66 | 67-68 | 69-70 | 71-72 | 73-74 | 75-77 | 78-89 |
|  | % total MWs^1^ | 11 | 10 | 12 | 12 | 9 | 10 | 10 | 9 | 9 | 9 |
|  | average score/MW | 125-183 | 183-194 | 194-201 | 201-206 | 206-212 | 212-218 | 218-227 | 227-236 | 236-248 | 248-304 |
|  | % total MWs | 10 | 10 | 10 | 10 | 10 | 10 | 10 | 10 | 10 | 10 |
| Zm_MF | % alignments/MW | 47-66 | 67-72 | 73-76 | 77-79 | 80-81 | 82-83 | 84-85 | 86-87 | 88-89 | 90-97 |
|  | % total MWs | 11 | 10 | 8 | 10 | 7 | 10 | 12 | 12 | 9 | 11 |
|  | average score/MW | 158-218 | 218-229 | 229-237 | 237-246 | 246- 255 | 255- 267 | 267-279 | 279-293 | 293-311 | 311-384 |
|  | % total MWs | 10 | 10 | 10 | 10 | 10 | 10 | 10 | 10 | 10 | 10 |
| Zm_TA | % alignments/MW | 43-71 | 72-76 | 77-79 | 80-82 | 83-84 | 85-86 | 87-88 | 89-90 | 91-92 | 93-99 |
|  | % total MWs | 8 | 8 | 7 | 9 | 10 | 11 | 12 | 12 | 10 | 12 |
|  | average score/MW | 264-424 | 424-457 | 457-475 | 475-492 | 492-507 | 507-522 | 522-535 | 535-552 | 552-579 | 579-686 |
|  | % total MWs | 10 | 10 | 10 | 10 | 10 | 10 | 10 | 10 | 10 | 10 |
| Hv_TA | % alignments/MW | 51-71 | 72-76 | 77-79 | 80-81 | 82-83 | 84-85 | 86-87 | 88 | 89-90 | 91-97 |
|  | % total MWs | 10 | 9 | 10 | 9 | 10 | 11 | 13 | 6 | 12 | 10 |
|  | average score/MW | 262-397 | 397-423 | 423-444 | 444-461 | 461-475 | 475-488 | 488-500 | 500-518 | 518-546 | 546-667 |
|  | % total MWs | 10 | 10 | 10 | 10 | 10 | 10 | 10 | 10 | 10 | 10 |
| Gm_TA | % alignments/MW | 7-19 | 20-22 | 23-24 | 25-26 | 27-28 | 29-30 | 31 | 32-33 | 34-35 | 36-47 |
|  | % total MWs | 10 | 10 | 10 | 11 | 13 | 13 | 6 | 10 | 9 | 8 |
|  | Average score/MW | 102-128 | 128-134 | 134-139 | 139-145 | 145-151 | 151-158 | 158-165 | 165-173 | 173-185 | 185-262 |
|  | % total MWs | 10 | 10 | 10 | 10 | 10 | 10 | 10 | 10 | 10 | 10 |
| At_TA | % alignments/MW | 5-14 | 15-16 | 17-18 | 19 | 20 | 21 | 22-23 | 24-25 | 26-27 | 28-37 |
|  | % total MWs | 9 | 8 | 11 | 8 | 8 | 8 | 17 | 13 | 9 | 10 |
|  | Average score/MW | 92-121 | 121-129 | 129-134 | 134-140 | 140-146 | 146-152 | 152-158 | 158-167 | 167-183 | 183-334 |
|  | % total MWs | 10 | 10 | 10 | 10 | 10 | 10 | 10 | 10 | 10 | 10 |
| Os_CD | % segmentally duplicated FAexpTRL/MW | 0 | 1-7 | 8-14 | 15-20 | 21-25 | 26-30 | 31-35 | 36-41 | 42-48 | 49-91 |
|  | % total MWs | 13 | 9 | 10 | 8 | 10 | 10 | 10 | 11 | 9 | 9 |
| Os_CD | Mean gene family size | 5.51-7.71 | 5.27-5.50 | 5.13-5.26 | 5.00-5.12 | 4.87- 4.99 | 4.75- 4.86 | 4.62-4.74 | 4.49-4.61 | 4.33-4.48 | 3.56-4.32 |
|  | % total MWs | 10 | 10 | 10 | 10 | 10 | 10 | 10 | 10 | 10 | 10 |

^1^% total MWs given to the nearest whole number

Additional file 2 Table 4. The relation of family size within the Os_CD database to MW number used in the calculation of MW average scores.

| Family size^1^ | No. FAexpTRL | % Total FAexpTRL | MW number |
| --- | --- | --- | --- |
| 1 | 3205 | 18.7 | 1 |
| 2 | 2210 | 12.9 | 2 |
| 3 | 1398 | 8.2 | 3 |
| 4-5 | 1586 | 9.3 | 4 |
| 6-8 | 1368 | 8.0 | 5 |
| 9-13 | 1353 | 7.9 | 6 |
| 14-25 | 1428 | 8.3 | 7 |
| 26-59 | 1623 | 9.5 | 8 |
| 60-218 | 1368 | 8.0 | 9 |
| 219-412 | 1569 | 9.2 | 10 |

^1^Family sizes are based on identical annotations of individual functionally annotated, expressed TIGR rice loci (FAexpTRL).
