# Supplementary material for: Rice pseudomolecule-anchored cross-species DNA sequence alignments indicate regional genomic variation in expressed sequence conservation
Source: BMC Genomics. 2007 Aug 20;8:283. doi: 10.1186/1471-2164-8-283 (PMC2041955; doi:10.1186/1471-2164-8-283)
Supplement: Additional file 3 — Illustrates the %MegaBLAST alignments/rice pseudomolecule between FAexpTRL and the plant databases. [file 1471-2164-8-283-S3.doc]

0

10

20

30

40

50

60

70

80

90

100

5

1

3

8

2

4

7

6

9

10

11

12

**Rice pseudomolecule**

**Percentage aligned FAexpTRL**

**Additional file 3. Sequence alignment distribution per rice pseudomolecule.** Percentage functionally annotated, expressed TIGR rice loci (FAexpTRL) from Os_CD database aligned with the test databases Lp_MF (purple), Zm_MF (red), Zm_TA (dark blue), Hv_TA (light blue), Gm_TA (yellow) and AT_TA (orange). Results are expressed per pseudomolecule.
