# Supplementary material for: Rice pseudomolecule-anchored cross-species DNA sequence alignments indicate regional genomic variation in expressed sequence conservation
Source: BMC Genomics. 2007 Aug 20;8:283. doi: 10.1186/1471-2164-8-283 (PMC2041955; doi:10.1186/1471-2164-8-283)
Supplement: Additional file 1 — Supplementary methods describing the derivation of FAexp gene family sizes based upon identical annotations. [file 1471-2164-8-283-S1.doc]

**Additional file 1**

Derivation of gene family sizes.

Supporting information is available in Additional files 2, 4 and 6.

FAexpTRL were sorted according to the exact annotation and the number of FAexpTRL for each identical annotation was derived. This identified 5602 different annotations which could be divided into 66 different family sizes ranging from 1 (3205 unique annotations) to 412 (protein kinase domain containing protein, expressed). The annotations were divided into 10 groups according to family size and each group was assigned a number, 1-10 (the MW number; see Additional file 2 Table 4 for details of family group sizes and MW numbers). Each FAexpTRL was assigned its MW number (1-10, relating its annotation to relative family size) and the mean number for each MW/100FAexpTRL was calculated for each pseudomolecule. The MW average numbers were colour coded according to Additional file 2 Table 3 and are illustrated in Additional file 4.
