# Supplementary material for: Rice pseudomolecule-anchored cross-species DNA sequence alignments indicate regional genomic variation in expressed sequence conservation
Source: BMC Genomics. 2007 Aug 20;8:283. doi: 10.1186/1471-2164-8-283 (PMC2041955; doi:10.1186/1471-2164-8-283)
Supplement: Additional file 4 — Illustrates colour coded MWs comparing %alignments between the Os_CD database and the test databases in relation to average gene family size. [file 1471-2164-8-283-S4.doc]

**1 2 3 4 5 6 7 8 9 10 11 12**

**Increasing %alignment / decreasing family size**


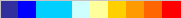

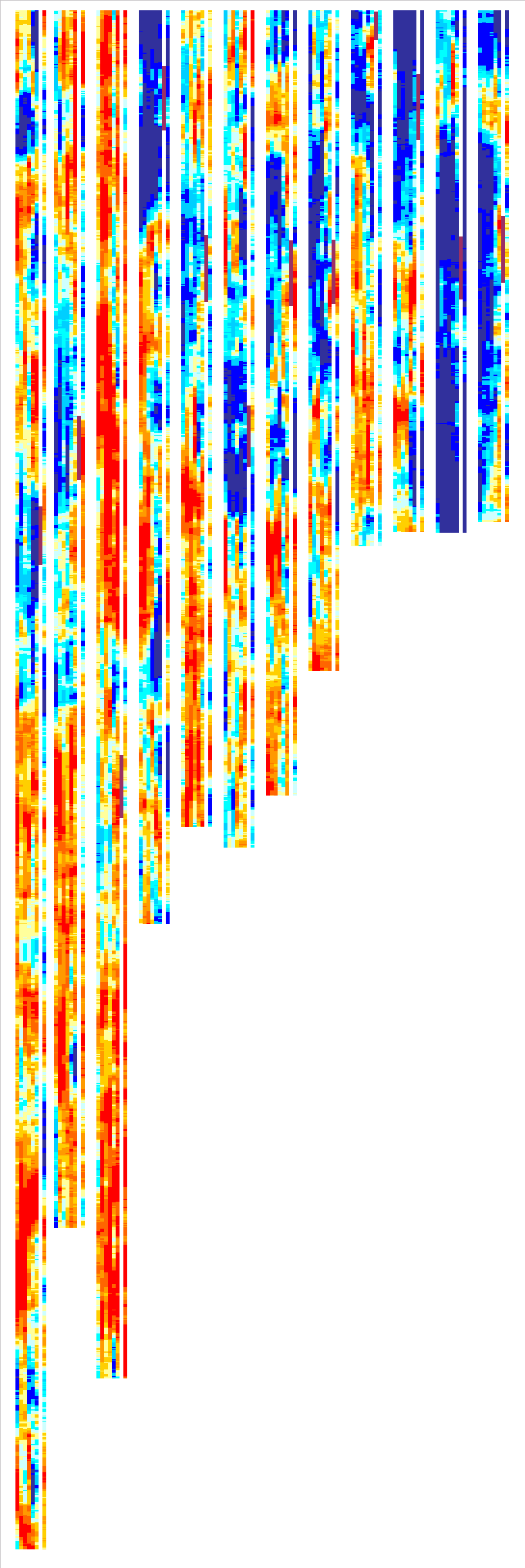

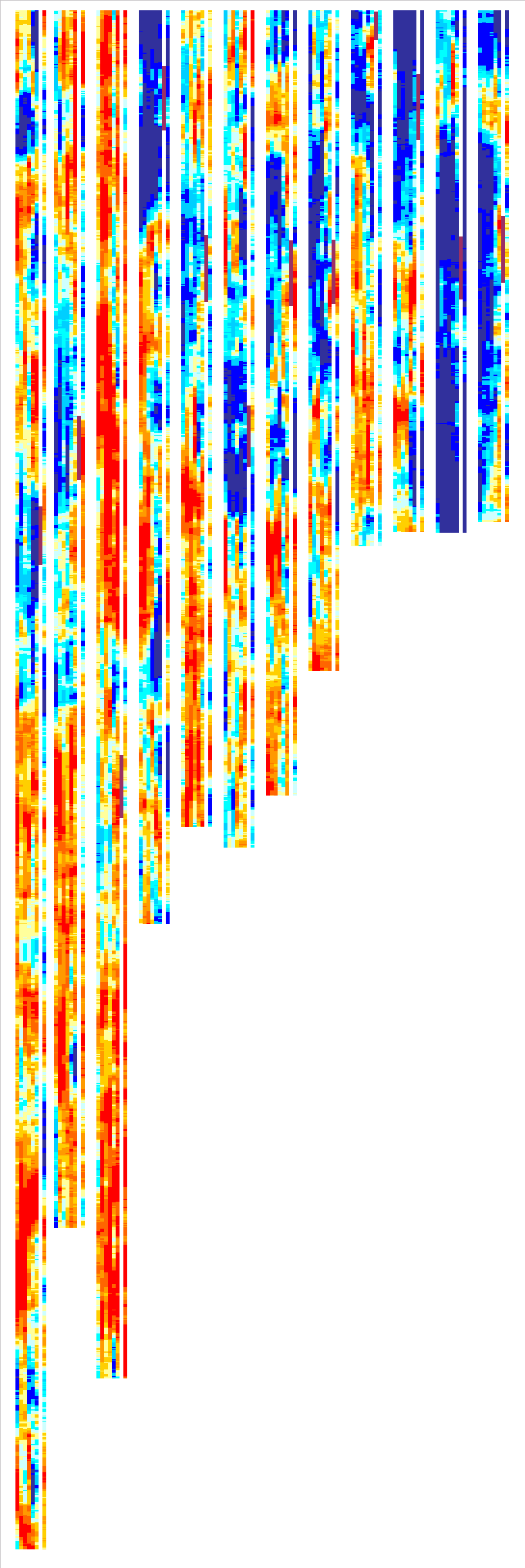

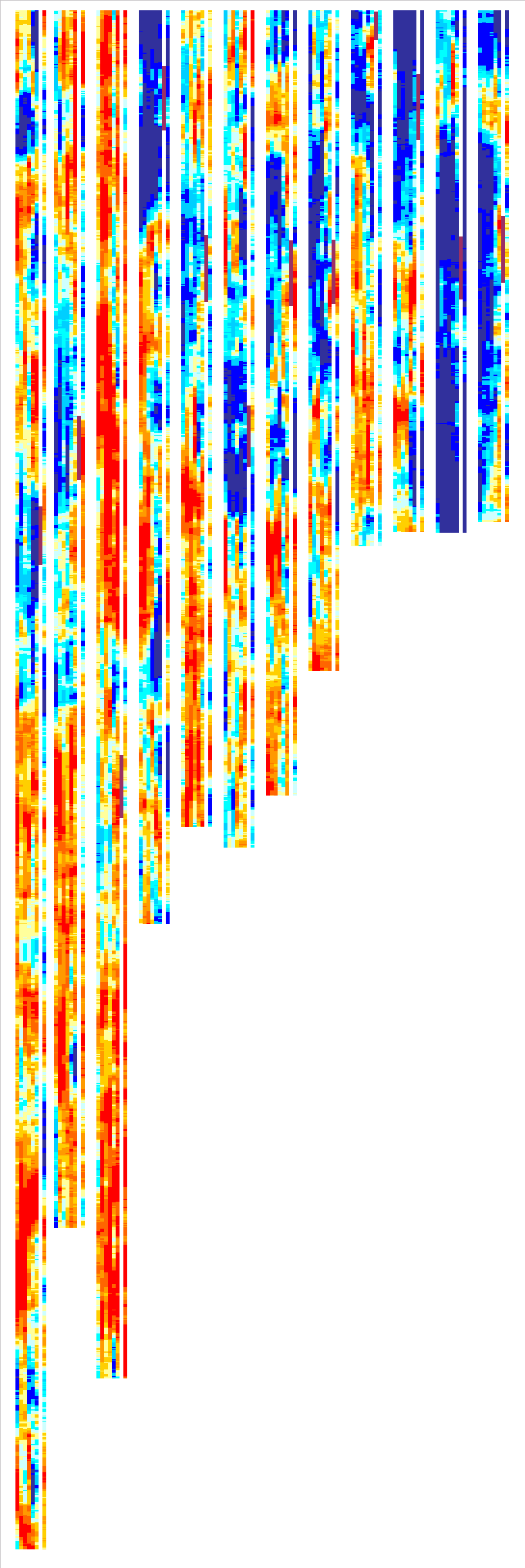

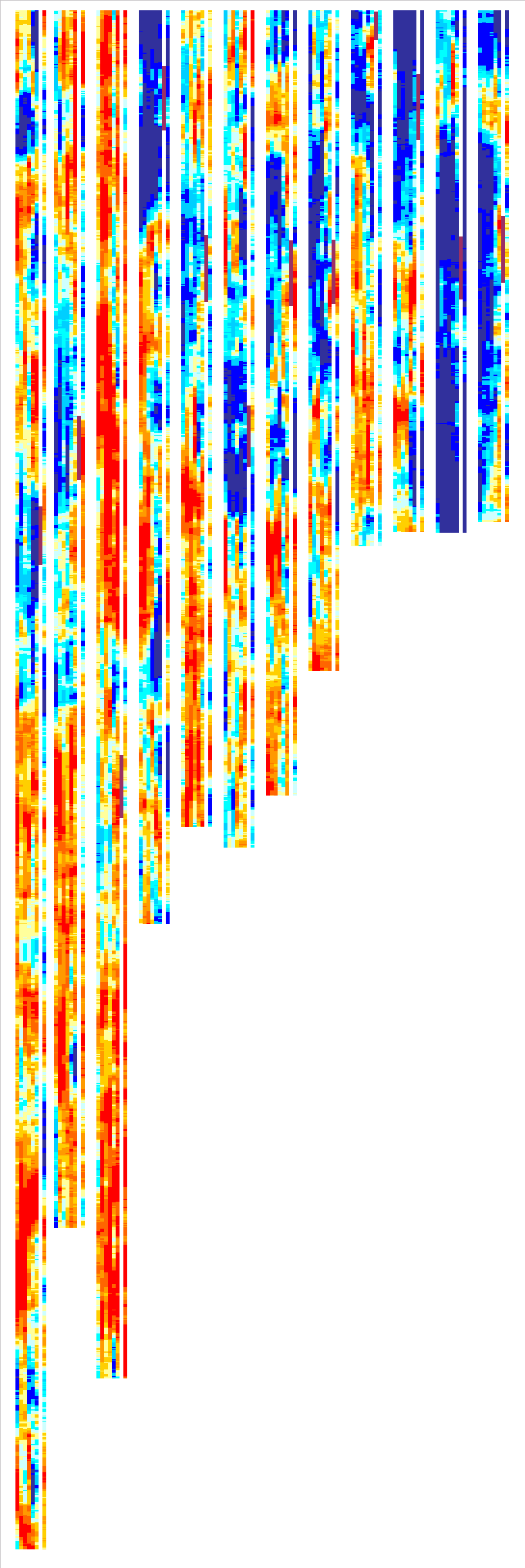

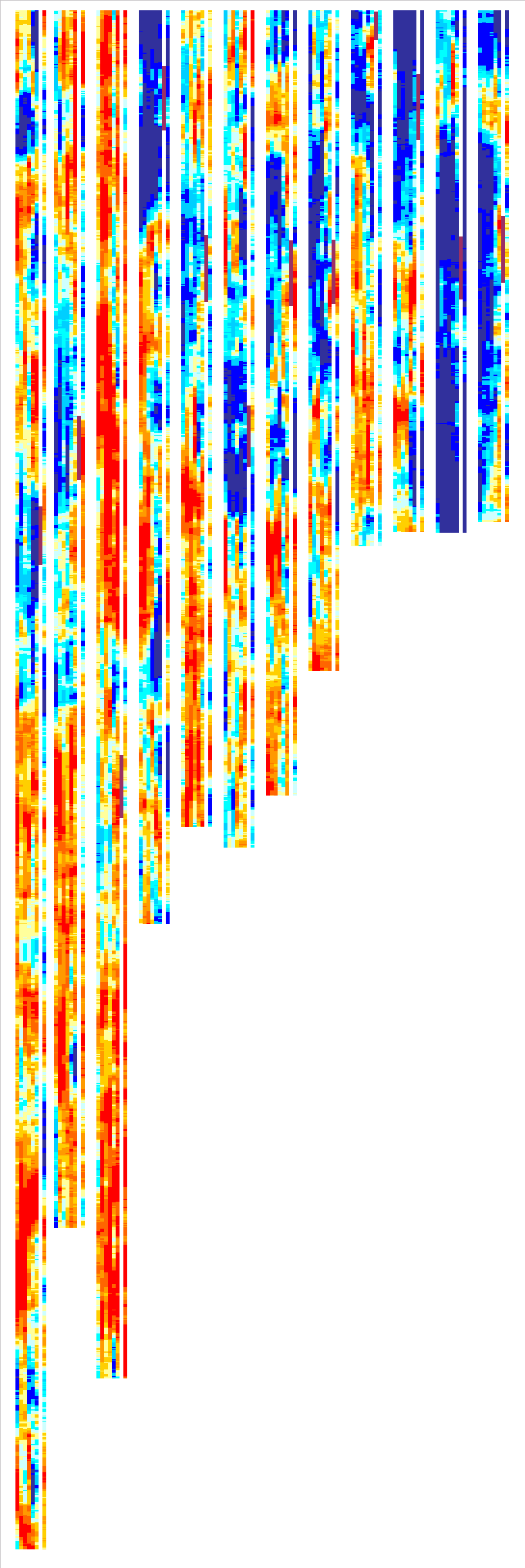

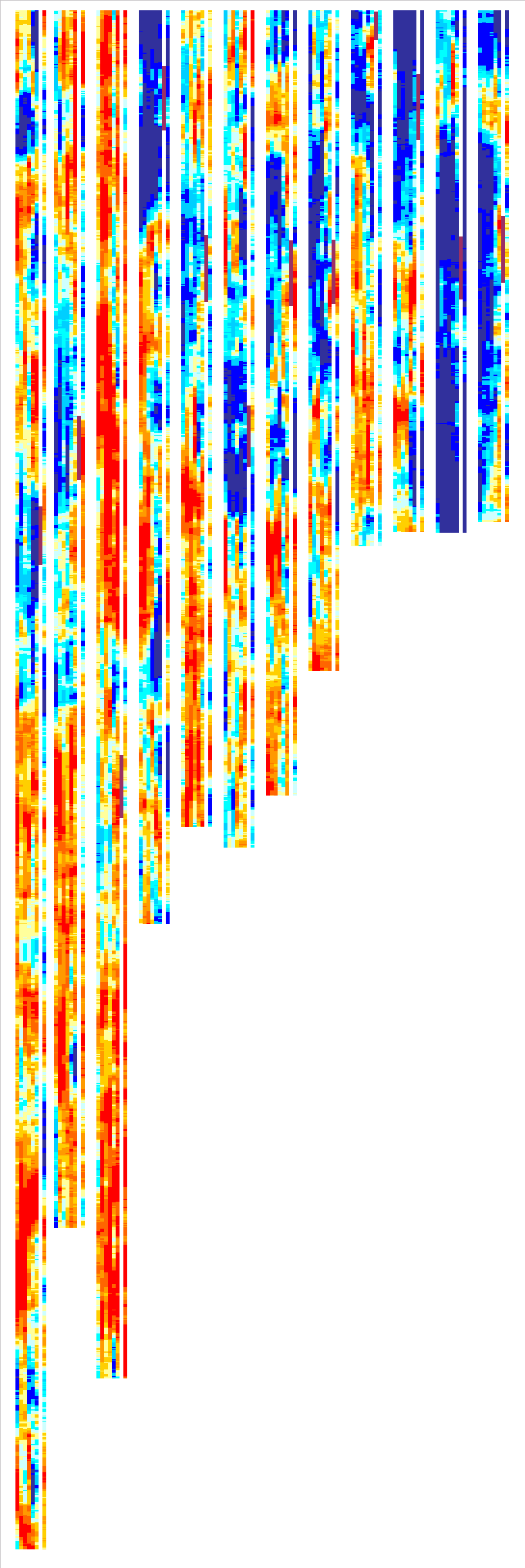

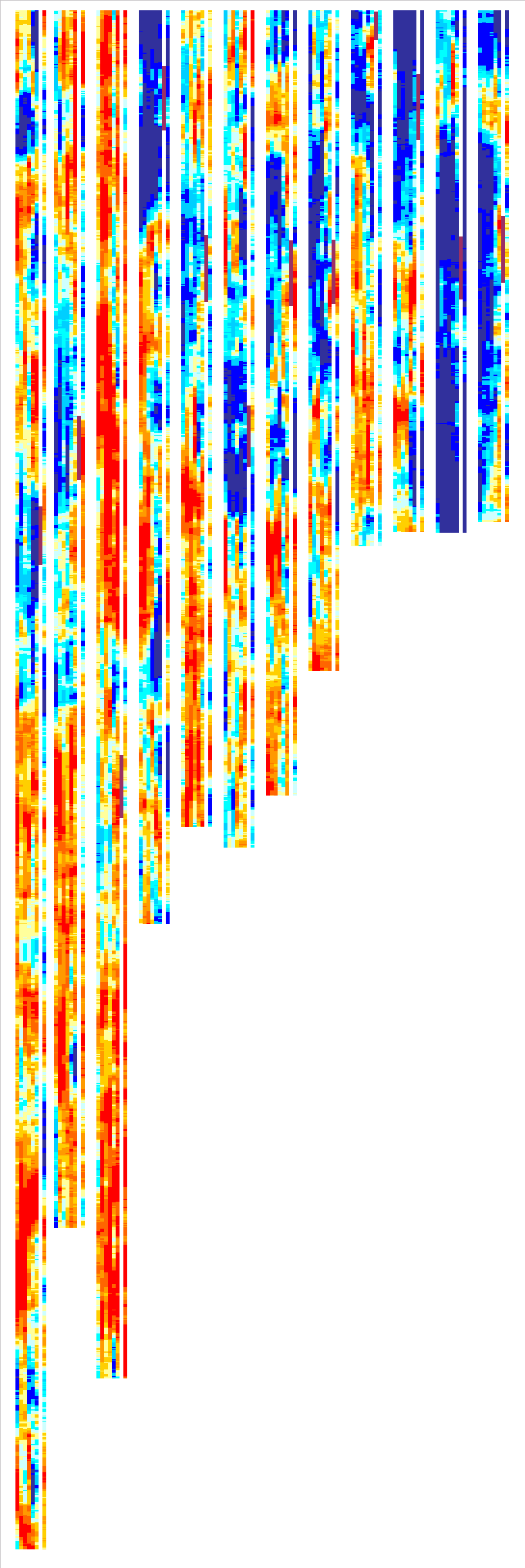

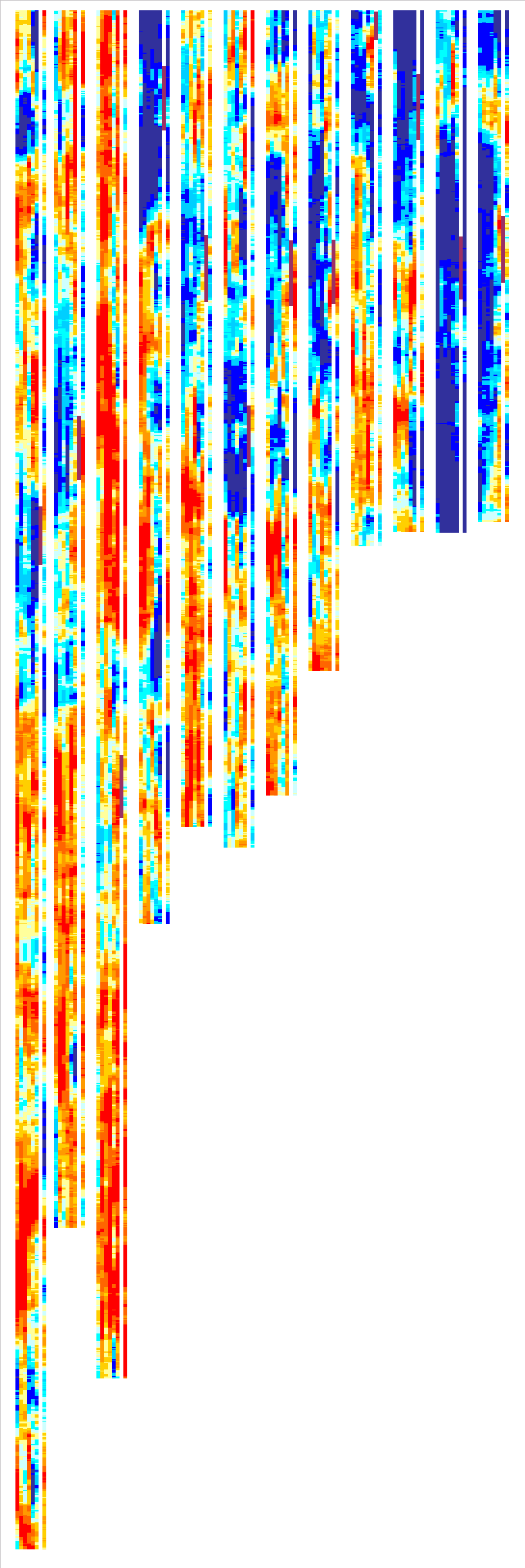

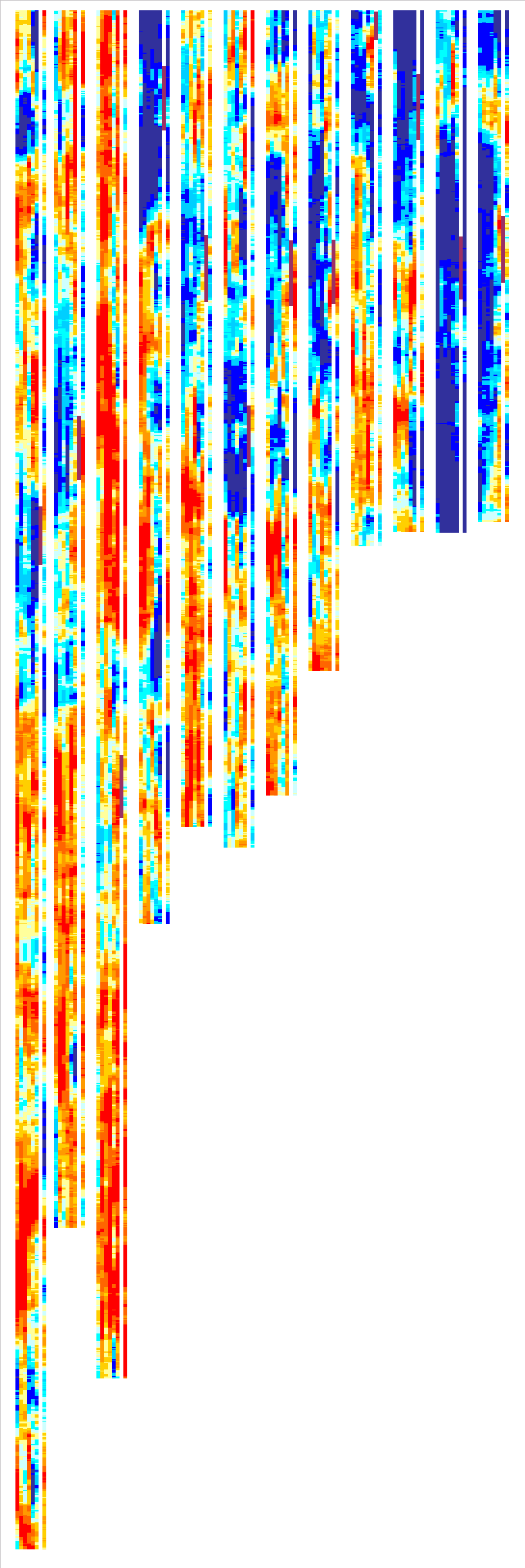

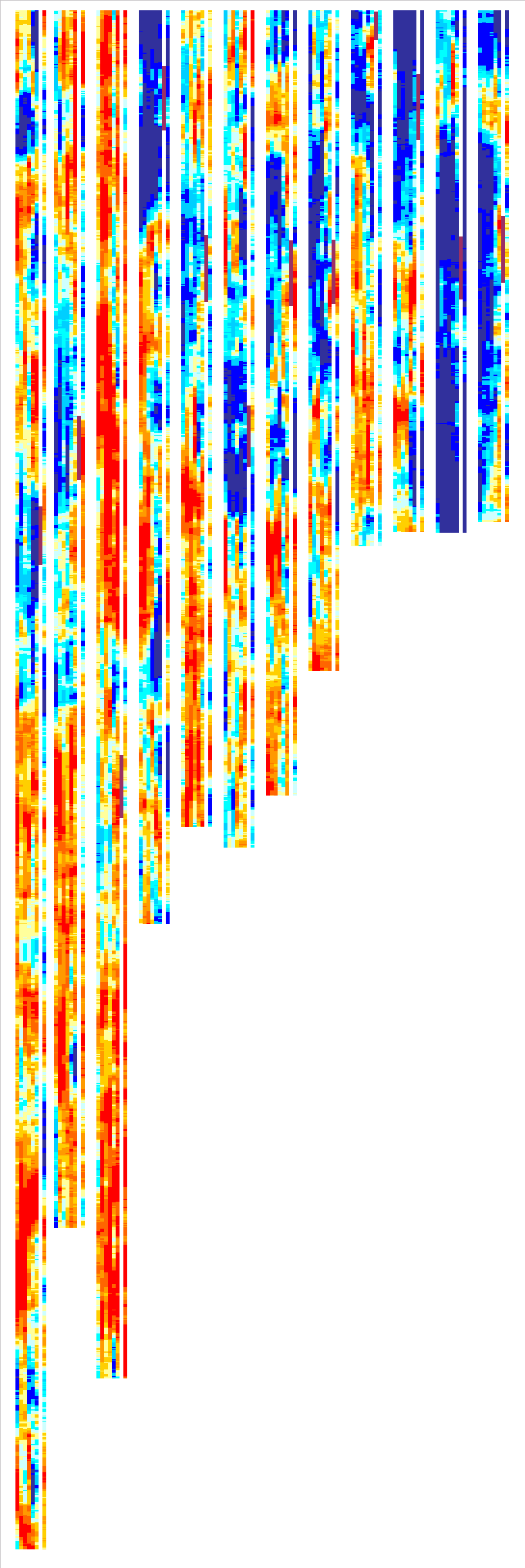

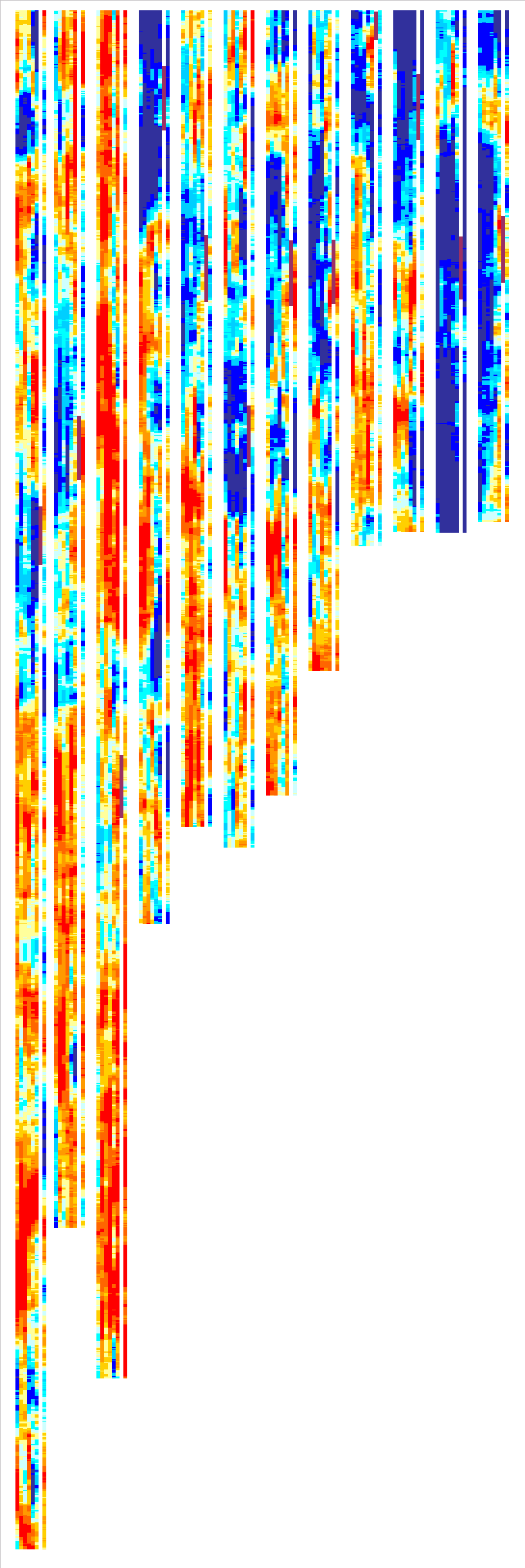

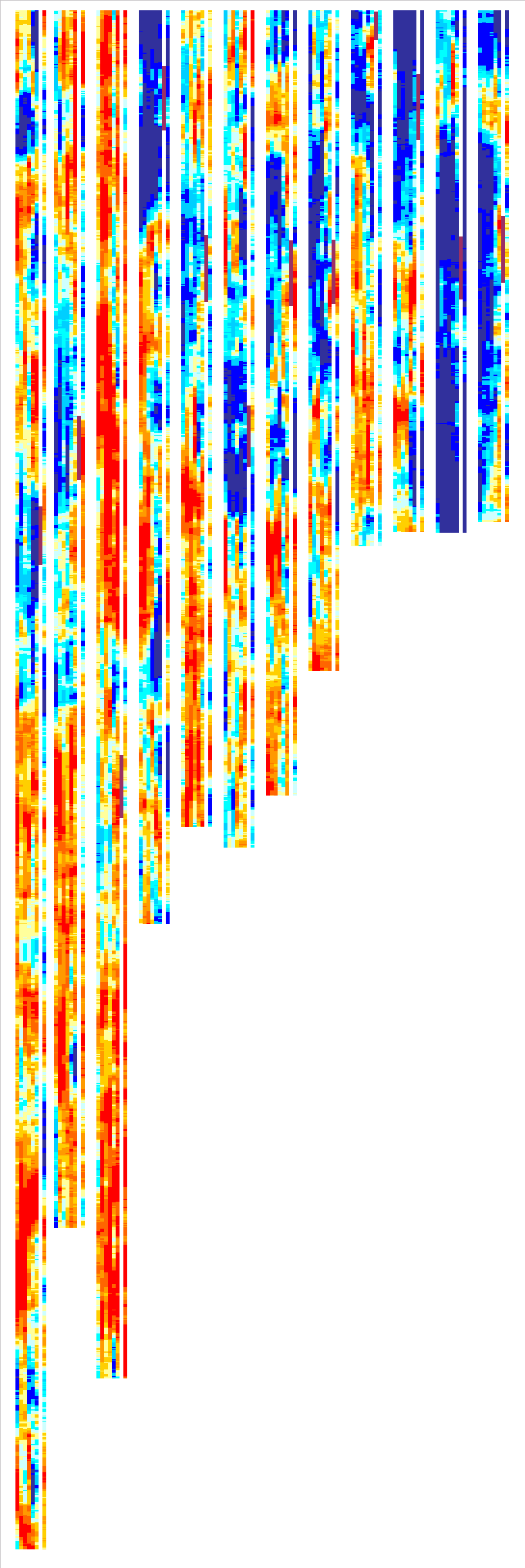


**Additional file 4. Heat maps for % sequence alignments and gene family sizes.** Colour coded moving windows/100 functionally annotated, expressed TIGR rice loci (MWs/FAexpTRL) for each rice pseudomolecule (1-12). For each pseudomolecule: column 1-6 = MWs for % significant MegaBLAST alignments between Os_CD and test databases Lp_MF, Zm_MF, Zm_TA, Hv_TA, Gm_TA and AT_TA, respectively; column 7 = position of MWs containing rice centromere (dark vertical bar); column 8 = MWs indicating the distribution of FAexpTRL from different sized gene families [see Additional file 2 Table 3 for colour code key and Additional file 1 for family size definitions].
